# Genomic analysis of field pennycress (*Thlaspi arvense*) provides insights into mechanisms of adaptation to high elevation

**DOI:** 10.1186/s12915-021-01079-0

**Published:** 2021-07-22

**Authors:** Yupeng Geng, Yabin Guan, La Qiong, Shugang Lu, Miao An, M. James C. Crabbe, Ji Qi, Fangqing Zhao, Qin Qiao, Ticao Zhang

**Affiliations:** 1 Yunnan Key Laboratory of Plant Reproductive Adaptation and Evolutionary Ecology, School of Ecology and Environmental Sciences, Yunnan University, Kunming, 650500 China; 2 School of Life Sciences, Yunnan University, Kunming, 650504 China; 3 Research Center for Ecology, College of Science, Tibet University, Lhasa, 850000 China; 4 Renji Hospital, School of Medicine, Shanghai Jiao Tong University, Shanghai, 200001 China; 5 Wolfson College, Oxford University, Oxford, OX2 6UD UK; 6 Institute of Biomedical and Environmental Science & Technology, School of Life Sciences, University of Bedfordshire, Park Square, Luton, LU1 3JU UK; 7 School of Life Sciences, Shanxi University, Taiyuan, 030006 China; 8 School of Life Sciences, Fudan University, Shanghai, 200433 China; 9 Beijing Institutes of Life Science, Chinese Academy of Sciences, Beijing, 100101 China; 10 University of Chinese Academy of Sciences, Beijing, 100049 China; 11 Center for Excellence in Animal Evolution and Genetics, Chinese Academy of Sciences, Kunming, 650223 China; 12 School of Agriculture, Yunnan University, Kunming, 650504 China; 13 College of Chinese Material Medica, Yunnan University of Chinese Medicine, Kunming, 650500 China

**Keywords:** Adaptive evolution, Transposable elements, Population genomics, FLOWERING LOCUS C, Qinghai-Tibet Plateau

## Abstract

**Background:**

Understanding how organisms evolve and adapt to extreme habitats is of crucial importance in evolutionary ecology. Altitude gradients are an important determinant of the distribution pattern and range of organisms due to distinct climate conditions at different altitudes. High-altitude regions often provide extreme environments including low temperature and oxygen concentration, poor soil, and strong levels of ultraviolet radiation, leading to very few plant species being able to populate elevation ranges greater than 4000 m. Field pennycress (*Thlaspi arvense*) is a valuable oilseed crop and emerging model plant distributed across an elevation range of nearly 4500 m. Here, we generate an improved genome assembly to understand how this species adapts to such different environments.

**Results:**

We sequenced and assembled de novo the chromosome-level pennycress genome of 527.3 Mb encoding 31,596 genes. Phylogenomic analyses based on 2495 single-copy genes revealed that pennycress is closely related to *Eutrema salsugineum* (estimated divergence 14.32–18.58 Mya), and both species form a sister clade to *Schrenkiella parvula* and genus *Brassica*. Field pennycress contains the highest percentage (70.19%) of transposable elements in all reported genomes of Brassicaceae, with the retrotransposon proliferation in the Middle Pleistocene being likely responsible for the expansion of genome size. Moreover, our analysis of 40 field pennycress samples in two high- and two low-elevation populations detected 1,256,971 high-quality single nucleotide polymorphisms. Using three complementary selection tests, we detected 130 candidate naturally selected genes in the Qinghai-Tibet Plateau (QTP) populations, some of which are involved in DNA repair and the ubiquitin system and potential candidates involved in high-altitude adaptation. Notably, we detected a single base mutation causing loss-of-function of the FLOWERING LOCUS C protein, responsible for the transition to early flowering in high-elevation populations.

**Conclusions:**

Our results provide a genome-wide perspective of how plants adapt to distinct environmental conditions across extreme elevation differences and the potential for further follow-up research with extensive data from additional populations and species.

**Supplementary Information:**

The online version contains supplementary material available at 10.1186/s12915-021-01079-0.

## Background

A cornerstone of biodiversity is that different species occupy specific ecological niches [1]. Each species has a range of distribution; some species are very narrow, and some can be widely distributed around the world [2, 3]. Altitude gradient, as an important index of spatial niche measurement, greatly affects the distribution range and pattern of organisms. Climate conditions vary greatly in different altitude regions [4]. Compared with low-altitude areas, high-altitude areas often have extreme environments including low temperature, low oxygen, poor soils, and strong ultraviolet (UV) radiation [5]. Only very few species can live across an elevation range greater than 4000 m from sea level to high altitude. Understanding how these species adapt to different environments with such a large altitude span can make a significant contribution to evolutionary ecology. It is difficult to demonstrate how wild species adapt to their local environments without genomics tools, because the mechanisms of ecological adaptation are very complex and involve multi-gene interactions at the genomic level rather than single-gene mutations [6, 7]. Previous genome-wide studies on adaptive evolution in distinct environments with great altitude differences have focused mainly on humans and vertebrates [8, 9]. Recently, several studies have reported the genetic architectures and evolutionary processes driving the adaption of alpine plants along altitudinal gradients [10–12]. However, local adaptation of alpine plants across the extreme altitude gradient (~ 4000 m) on the Qinghai-Tibet Plateau (QTP) is very scarce.

Field pennycress (*Thlaspi arvense* L.) is an annual diploid (2n = 2x = 14) valuable oilseed crop and vegetable as well as a farmland weed of Brassicaceae. In recent years, this plant has attracted great attention, since pennycress is a biofuel feedstock crop (pennycress seeds contain an average of 33% oil by weight) and possesses important agronomic traits [13–17]. Moreover, pennycress can reproduce from one generation to the next by seed in less than 10 weeks, and transgenic protocols for *Arabidopsis thaliana* have also worked well for pennycress, making it a supplemental model system to *A*. *thaliana* [14, 15]. Field pennycress is widely distributed in temperate regions of the Northern Hemisphere and usually grows in habitats such as roadsides, fields, and grassy slopes. It has been reported that field pennycress is mainly self-pollinated because its cleistogamous development (pollination occurring inside a closed flower) of flowers minimizes outcrossing [18]. There are both winter (late) and spring (early) flowering habits of pennycress in wild populations [17–20]. What is more significant is that this plant can live in diverse environments from sea level (late flowering) to nearly 4500 m (early flowering) on the QTP in China [20, 21]. Our previous molecular dating study suggested that specific haplotypes of field pennycress on the QTP diverged around 1.58 Mya, which corresponds to the Qingzang Movement of the QTP uplift [21]. The QTP has been considered the third pole on Earth because of the harsh environment [5]. How field pennycress has adapted and evolved to the highly heterogenous environments from sea level to highland remains unclear, and this plant should be an ideal system for the study of ecological adaptation in distinct environments.

A draft genome sequence of field pennycress has been released with a total assembly length of 343.01 Mb (scaffold N50 = 0.14 Mb), only accounting for 63.63% of the previously predicted genome size of 539 Mb [16], due to sequencing technology limitations. In the present study, by using Illumina Hiseq, Oxford Nanopore, and Hi-C (chromosome conformation capture) sequencing technologies, we provide a de novo high-quality chromosome-level genome sequence of field pennycress. Genome comparisons between pennycress and related species were conducted. We also have detected genome-wide single nucleotide polymorphisms (SNPs) associated with altitude variation in different populations of field pennycress. Finally, our study has identified several biological processes and related genes implicated for adaptation to distinct environments across nearly 4500 m of elevation difference. These findings provide novel insights into the genetic mechanisms of plant adaptation to the high altitude on the QTP.

## Results and discussion

### Genome assembly and annotation

**Fig. 1.**
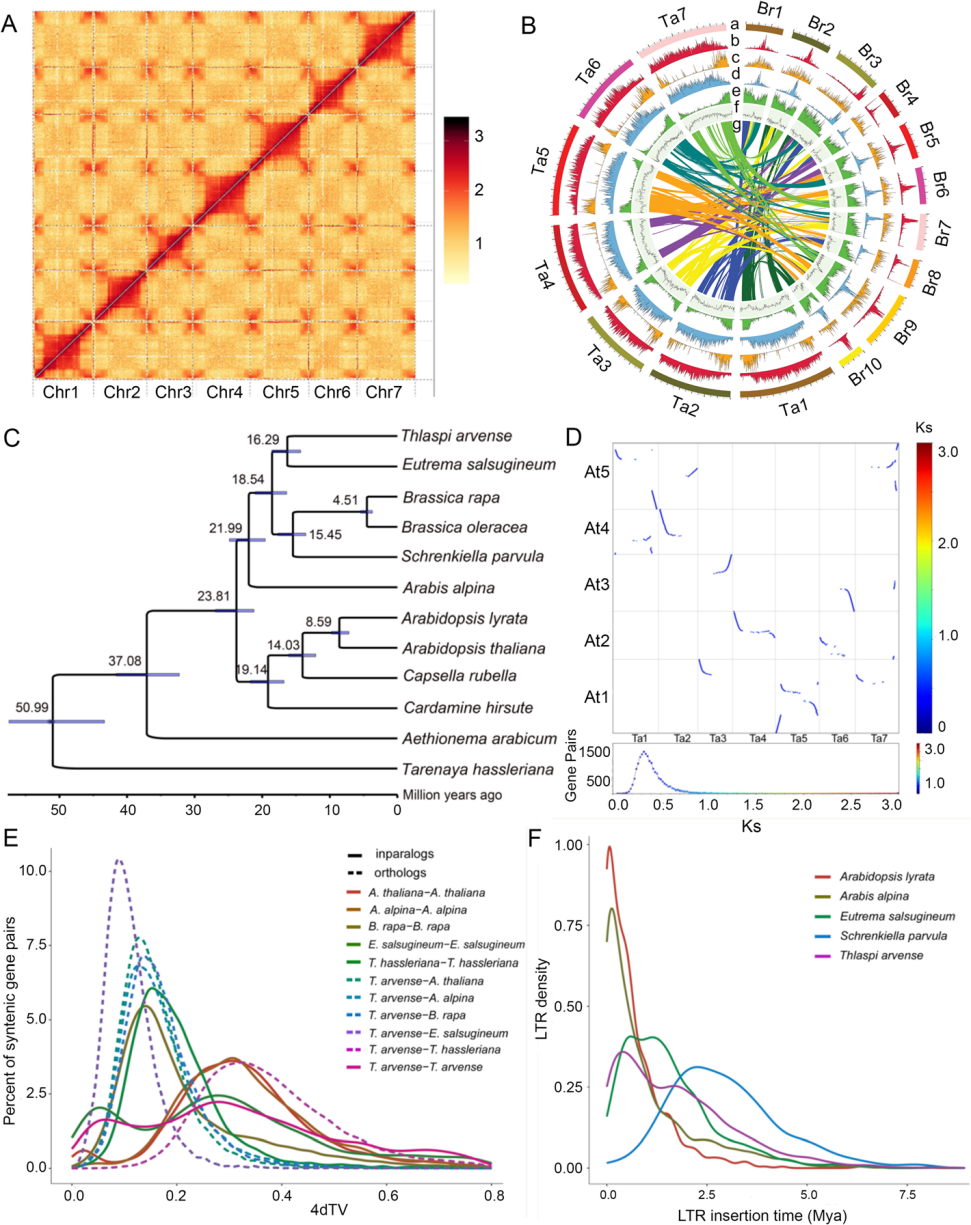
Comparative genomic analyses of field pennycress with relatives. **a** Hi-C interaction heatmap for pennycress genome showing interactions among seven chromosomes (Chr1–7). **b** Genomic features of pennycress (Ta) vs. *Brassica rapa* (Br). Tracks from outside to inside (a–g) are as follows: chromosomes, retrotransposon density, DNA transposon density, long terminal repeat retrotransposon (LTR) density, gene density, GC content, and collinearity between both genomes. **c** Maximum likelihood tree and estimation of divergence times in Brassicaceae. **d** Genome collinearity dot plot and Ks distribution between pennycress (Ta) and *A. thaliana* (At). **e** Age distribution of transversion substitutions at fourfold degenerate sites (4DTv) distance values between orthologs of pennycress and its relatives. **f** Insertion time distribution of LTR of pennycress and its relatives

The genome size of field pennycress was estimated to be 548.21 Mb with a low heterozygosity of 0.07% based on *k*-mer statistics [22] (Additional files 1, 2: Figure S1, Table S1). In total, we generated 76.62 Gb of short reads using an Illumina Hiseq platform and 55.26 Gb of long reads (mean read length = 21,926 bp) using Oxford Nanopore sequencing technology (Table 1). A genome assembly of 527.15 Mb, consisting of 3790 contigs (contig N50 = 4.18 Mb), was achieved by a combination of the Illumina and Nanopore reads. Furthermore, using 60.97 Gb of Hi-C clean data, 474.97 Mb (90.07% of the final assembled 527.3 Mb genome) of the contig sequences was anchored to seven pseudo-chromosomes (Table 1, Fig. 1a). The length of pseudo-chromosomes ranged from 57.5 to 75.83 Mb (scaffold N50 = 70.79 Mb) (Additional file 3: Table S2). A total of 31,596 protein-coding genes were predicted, including 31,026 (98.2%) functionally annotated genes (Table 1; Additional files 4, 5, 6: Tables S3-S5). The spatial distribution of these protein-coding genes along the chromosome was uneven with higher densities located at the ends of the chromosomal arms (Fig. 1a). The completeness of gene prediction was further assessed using Benchmarking Universal Single-Copy Orthologs (BUSCO) [23], which showed that 95.9% of the 1440 plant single-copy orthologs were complete (Additional file 7: Table S6).

**Table 1:** Genome assembly and annotation of field pennycress

Genome features	Count
Illumina PE150 reads (Gb)	76.62
Nanopore reads (Gb)	55.26
Hi-C reads (Gb)	60.97
Total length of contigs (Mb)	527.15
Total number of contigs	3790
Longest length of contigs (Mb)	22.18
Length of contig N50 (Mb)	4.18
Number of contig N50	24
Total assembly size (Mb)	527.3
Total anchored size (Mb)	474.97
Total number of scaffolds	2298
Longest length of scaffolds (Mb)	75.83
Length of scaffold N50 (Mb)	70.79
Number of scaffolds N50	4
GC content (%)	39.03
Repeat content (%)	70.19
BUSCO assessment (%)	95.9%
Number of predicted genes	31,596

### Proliferation of transposable elements

The field pennycress genome (assembled genome size of 527.3 Mb, estimated size of 548.2 Mb) is two or three times larger than closely related diploid species, such as *Eutrema salsugineum* (assembled genome size of 233.7 Mb, estimated size of ~ 260 Mb) [24] and *Schrenkiella parvula* (assembled genome size of 137.1 Mb, estimated size of ~ 160 Mb) [25]. Polyploidization (whole-genome duplication (WGD)) events and transposable element (TE) amplification are two major causes of genome expansion [26, 27]. Analysis of age distributions built from transversion substitutions at fourfold degenerate sites (4DTv) indicated that, except for the α (4DTv distance = ~ 0.3) polyploidy events, which are shared among the members of Brassicaceae [28], field pennycress has not undergone an additional species-specific whole-genome duplication (WGD) event. This result was also supported by syntenic dot plot analysis between field pennycress and *A. thaliana* as well as *E. salsugineum*; both reveal one-to-one orthologous syntenic blocks of genomic regions (Fig. 1d, Additional file 8: Figure S2). Comparison between field pennycress and *Brassica oleracea* showed one-to-three matchings (Additional file 8: Figure S2), which was consistent with previous reports that whole-genome triplication has occurred in *B. oleracea* since their divergence from the *Arabidopsis* lineage [29]. Therefore, based on the 4DTv and syntenic dot plot results, WGD as a possible mechanism causing expansion of field pennycress genome size could be excluded. Next, we investigated the content and evolutionary history of TEs in the field pennycress. Using de novo prediction of TEs (see the “Methods” section), we identified 370.13 Mb TE sequences and masked 70.19% of the genome assembly length (Additional file 9: Table S7), which is one of the highest percentages reported in all species of Brassicaceae (Additional file 10: Table S8). The highest proportion of TEs in field pennycress was long terminal repeat retrotransposons (LTR-RTs) (62.13%) (Additional file 9: Table S7). The retrotransposon proliferation is largely responsible for the expansion of genome size in field pennycress. Moreover, the LTR-RTs are mainly distributed in the large-scale region at the middle of chromosomes of field pennycress, resulting in the protein-coding genes mostly located at both ends of the chromosomal arms (Fig. 1a). This pattern was also identified in the genomes of *E. salsuginea* and wintersweet and reported due to stress-induced activation of LTR-RTs [24, 30]. To investigate the evolutionary dynamics of LTR retrotransposons, we estimated their approximate insertion times in five related species (Fig. 1e). Most complete LTR-RTs (3541) were identified in field pennycress, followed by 2993, 1699, 1327, and 85 LTR-RTs identified in *Arabis alpina*, *E. salsugineum*, *Arabidopsis lyrata*, and *S. parvula*, respectively, suggesting LTR-RTs still possessing transposition ability are most abundant in field pennycress and least abundant in *S. parvula*. Unlike *A. lyrata* and *A. alpina*, which had a comparatively higher proportion of recent insertions, the proliferation of LTR-RTs in field pennycress peaks at approximately ~ 0.5 Mya but continued within the past five million years, similar to *E. salsugineum*, while being younger than that of *S. parvula* (Fig. 1e). This suggests that the evolutionary dynamics and mobile activity of LTR-RTs of the five species varied over the last few million years.

### Phylogenetic tree and divergence times

Applying OrthoFinder2 [31] to twelve reported whole-genome sequences from Brassicaceae, we identified a total of 18,343 orthogroups. To verify the phylogenetic position of field pennycress, we generated a maximum likelihood phylogenetic tree with a trimmed and concatenated protein sequence alignment from 2495 single-copy genes in twelve species. The resulted phylogeny indicated that field pennycress was most closely related to *Eutrema salsugineum* (formerly *Thellungiella salsuginea*), and these two species in turn formed a clade with *Schrenkiella parvula* (formerly *Thellungiella parvula*), *Brassica rapa*, and *B. oleracea* (Fig. 1b). The abovementioned four genera, together with the allied *Arabis alpina*, were often recognized as lineage II or clade B in previous phylogenetic studies [32, 33]. Using MCMCtree [34] with four calibration points (see the “Methods” section, Fig. 1b), pennycress and *E. salsugineum* were estimated to have diverged approximately 16.28 Mya (95% confidence interval, 14.32–18.58 Mya); the two species diverged from the *Brassica* clade approximately 18.54 (16.39–21.04 Mya) and from the *Arabidopsis* clade approximately 23.81 (21.22–26.9 Mya). These dating results agree with previous estimates [35–37].

### Population genetic structure and demographic history

**Fig. 2.**
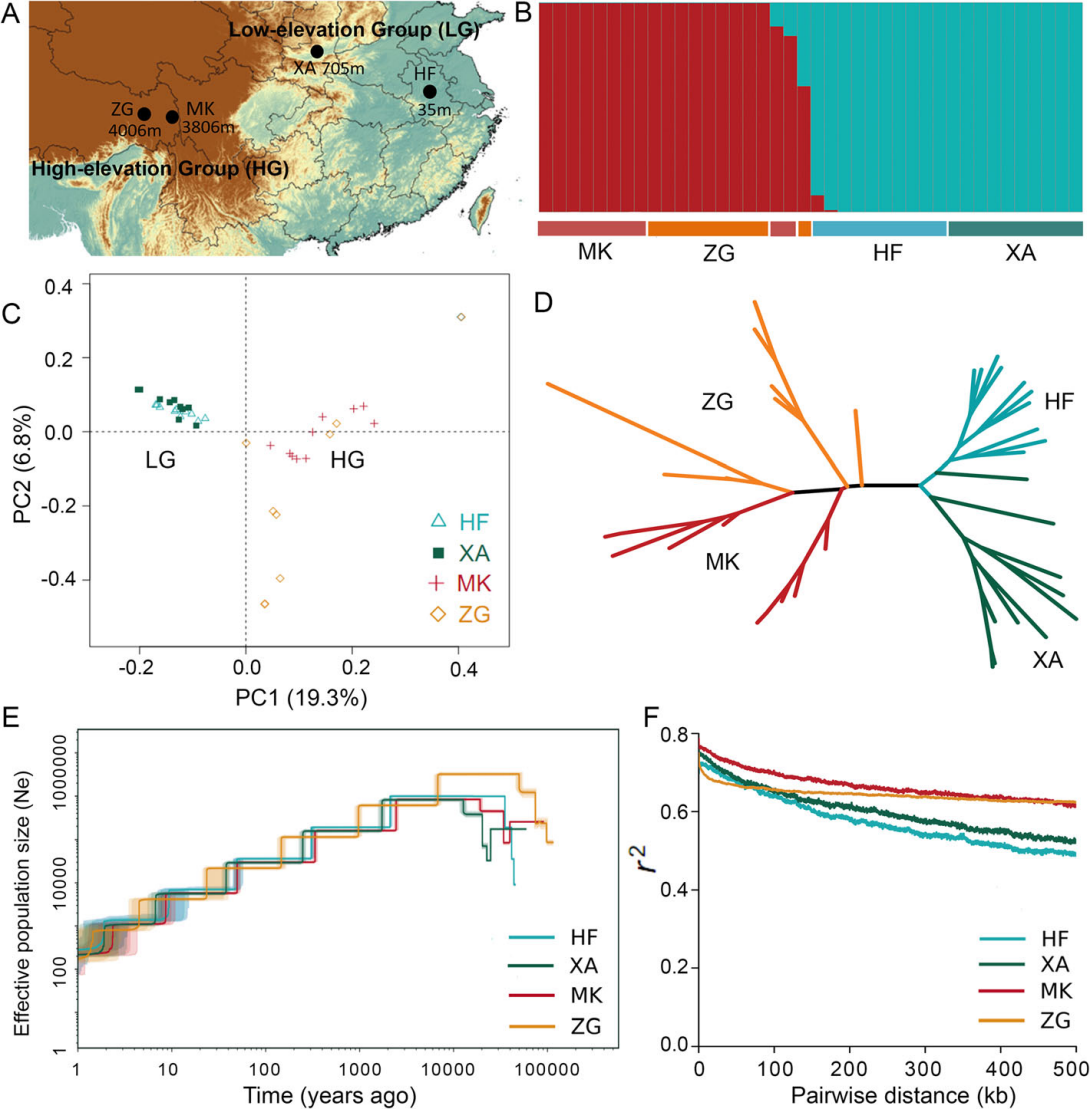
Population genetic structure and demographic history of field pennycress. **a** Sampling locations. For four site names and two groups, refer to Table 2. **b** Population structure plots with the number of ancestral clusters (K) = 2. **c** Principal component analysis (PCA) plot of all samples in four populations. **d** Maximum likelihood (ML) tree of all samples based on high-quality single nucleotide polymorphisms (SNPs). **e** Effective population size inferred based on SNPs for four populations. **f** Linkage disequilibrium (LD) patterns for the four pennycress populations. X-axis: physical distances between two SNPs marked in kb; Y-axis: R^2^ used to measure linkage disequilibrium

We re-sequenced 40 samples of four areas to identify differentiated genomic regions between two high- (MK and ZG) and two low-elevation (HF and XA) populations of field pennycress (Fig. 2a). We generated a total of 401.05 Gb of clean short reads after quality filtering in four populations (Table 2). Among them, 98.6% of reads can be mapped to our field pennycress reference genome in the present study (Additional file 11: Table S9), further demonstrating the high quality and completeness of the assembled genome. After mapping, ~ 19-fold average coverage depth per sample was estimated. Using a set of stringent criteria (see the “Methods” section), we identified 1,256,971 high-quality single nucleotide polymorphisms (SNPs) from 2,522,107 initially detected SNPs in all populations. We used the ADMIXTURE software [38] to infer the most likely number of ancestral groups and the four populations divided into two distinct ancestry clusters, a high-elevation group (HG) and a low-elevation group (LG) (Fig. 2b). A similar finding regarding genetic structure was further confirmed by the neighbor-joining (NJ) tree (Fig. 2c) and the principal component analysis (PCA) at PC1 axis (9.3%) according to altitude difference (Fig. 2d). The pairwise F_ST_ between populations ranged from 0.0816 to 0.2832, and the most genetic differentiation was found between HF and MK (Additional file 12: Table S10).

**Table 2:** Sampling locations, bio-climatic characterization, and genetic differentiation of field pennycress populations

Groups	Pop.	Lat. (N)	Lon. (E)	Alt. (m)	MTWQ (°C)	PWQ (mm)	Tajima’s D	θ_π_	θ_W_	Fst
LG	HF	31.924	117.139	35	27.25	394	0.6521	0.00042	0.00027	0.1818
	XA	34.022	109.116	705	24.45	286	0.7578			
HG	MK	29.658	98.565	3806	10.57	317	0.7188	0.00057	0.00045	
	ZG	29.956	97.412	4006	10.78	336	0.4953			

*LG* low-elevation group, *HG* high-elevation group, *Pop* populations, *Lat* latitude, *Lon* longitude, *Alt* altitude, *MTWQ* mean temperature of the warmest quarter, *PWQ* precipitation of the warmest quarter

We employed the Stairway plot [39] method to examine the changes in effective population size (Ne) of the four ancestral populations of field pennycress in response to Quaternary climatic change. The ancestral Ne of field pennycress showed continued declines in four populations (Fig. 2e). The Tajima’s D estimate also produced positive results for all four populations, suggesting field pennycress has not experienced recent population expansion (Table 2). It is consistent with our previous study of ecological niche modeling (ENM) both at the present time and at the last glacial maximum (LGM) based on the field pennycress specimen records (www.gbif.org) and world climate (www.worldclim.org/) information [21]. Our results indicated that there were widely suitable habitats in East China at the LGM (~ 20,000 years ago) rather than at present.

The patterns of the linkage disequilibrium (LD) observed in natural populations will be affected by factors such as non-random mating, selection, genetic drift, and the effective population size [40]. In our study, both HG and LG populations showed strong LD across the whole genome and a slow LD decay pattern (Fig. 2f, Additional file 13: Figure S3). The slow LD decay pattern may be caused by continued declining effective population size in field pennycress as has been reported in South African sheep [41]. Furthermore, LD decays occur more rapidly in cross-pollinated species as compared to self-pollinated species because recombination is less effective in the latter [42]. Therefore, the slow LD decay pattern also could be caused by the self-fertilizing mating system of field pennycress, which results in a slower breakup of LD blocks. In this study, the correlation coefficient of linkage disequilibrium (r^2^) of two high-altitude populations (ZG and MK) was relatively higher than that in two low-altitude populations (HF and XA) after ~ 150 kb (Fig. 2f, Additional file 13: Figure S3). This difference may reflect the variation in selfing rates between low- and high-elevation populations, but further experimental verification is needed. In addition, it has also been reported that the LD value of the natural selected or domesticated populations will be higher due to the positive selection effect [43]. Therefore, the difference in LD decay may also suggest that positive selection has occurred in high-elevation populations.

### Candidate positively selected genes (PSGs) in high-elevation groups

**Fig. 3.**
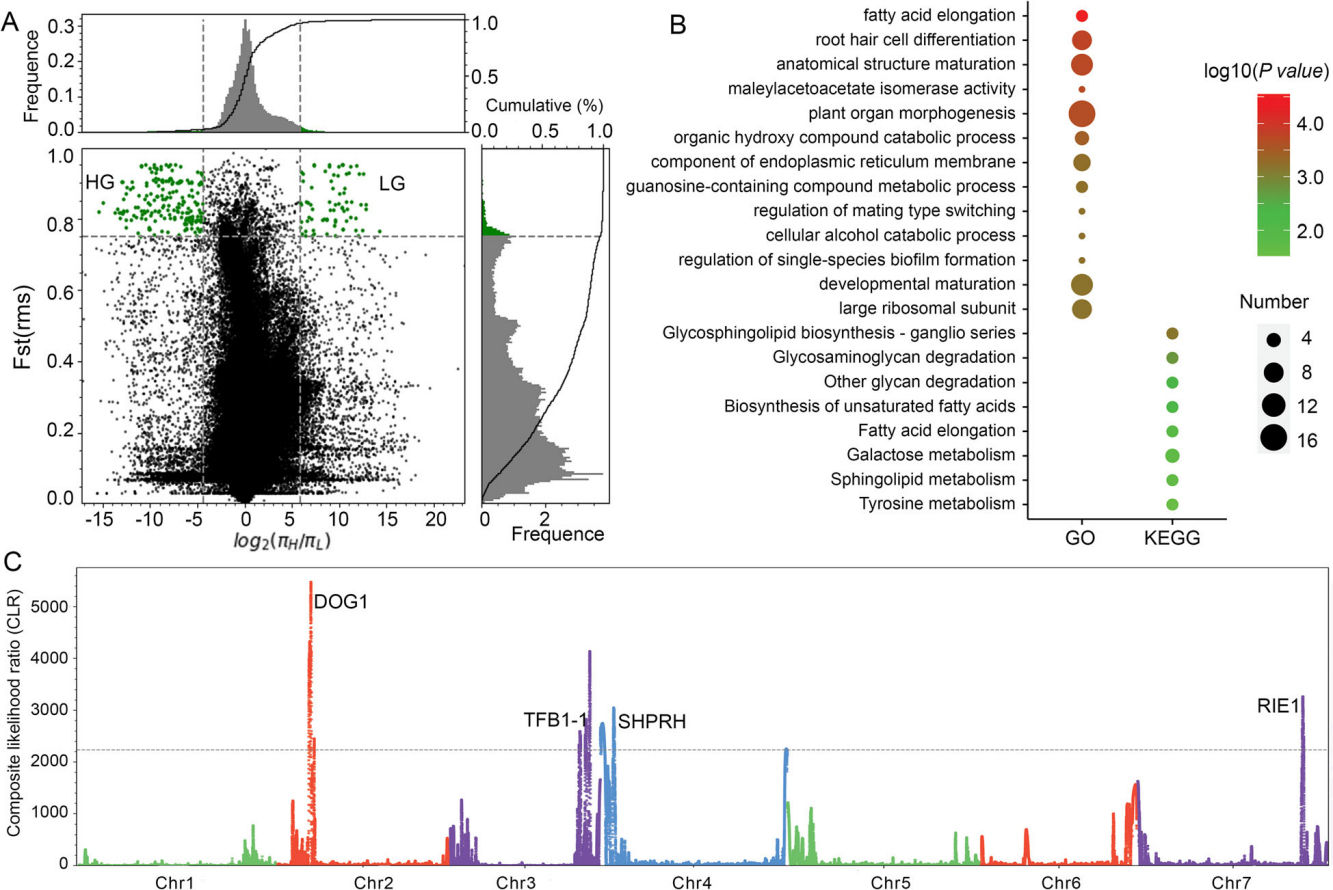
Candidate positively selected genes (PSGs) in high-elevation groups. **a** The distribution of the Fst and θ_π_ values in HG and LG. The vertical and horizontal dashed lines correspond to the 3% right tails of the Fst and θ_π_ value distribution, respectively. **b** Gene Ontology (GO) and KEGG functional classification of candidate selected genes in HG. **c** The composite likelihood ratio (CLR) result in the HG using the top 1% percentile outlier threshold

Genes under natural selection are usually assumed to be in highly differentiated genomic regions, which can be measured by the nucleotide heterozygosity (θ_π_) and the F statistic (F_ST_) [43, 44]. The θ_π_ value of HG (0.00057) is higher than that of LG (0.00042), and the F_ST_ value between HG and LG is 0.1818, suggesting significant genetic differentiation occurred between these two regions (Table 2). Selective sweep analysis by combination of F_ST_ and θ_π_ ratio has proved to be an effective method to detect natural selection signals related to the living environment [44]. We used a top 3% cutoff of both root mean square (rms) of F_ST_ [F_ST_(rms)] and θ_π_ ratio for highly differentiated SNPs and identified 359 candidate PSGs in HG and 165 PSGs in LG (Fig. 3a, Additional file 14: Table S11). The GO annotation was conducted and the most significantly enriched terms included “fatty acid elongation” (3 PSGs, *P* value = 2.85E−5) and “root hair cell differentiation” (8 PSGs, *P* value = 0.00016), (Fig. 3b, Additional file 15: Tables S12). Moreover, we conducted the composite likelihood ratio (CLR) test in the HG using the top 1% percentile outlier threshold, which detected 621 PSGs. The most significantly enriched GO terms included “positive regulation of secondary metabolite biosynthetic process” (3 PSGs, *P* value = 0.00061), “positive regulation of stress-activated MAPK cascade” (3 PSGs, *P* value = 0.0012), and “response to UV-C” (4 PSGs, *P* value = 0.0014) (Additional file 16: Tables S13). KEGG classification of both F_ST_(rms) and θ_π_ as well as CLR analysis showed significant enrichment in “Glycan degradation and biosynthesis” (Fig. 3b).

It is noteworthy that the combination of these three complementary selection tests [F_ST_(rms), θ_π_ ratio, and CLR] detected 130 overlapped PSGs in HG (Additional file 17: Tables S14), which should be credible candidate genes to adapt to a high-altitude extreme environment. These genes are mainly located on the adjacent region of chromosomes 2, 3, 4, and 7, which may be caused by natural selection along with genetic hitchhiking effects [7]. A major stress factor associated with high-altitude conditions is the increase in solar radiation intensity, inducing significant levels of DNA damage in living organisms. Accordingly, several of the most differentiated SNPs in field pennycress populations are in DNA repair- and ubiquitin pathway-related genes. These include general transcription and DNA repair factor IIH subunit TFB1-1 (Ta.Chr3.4008) which is involved in nucleotide excision repair of damaged DNA and tandemly duplicated paralogs E3 ubiquitin-protein ligase SHPRH (Ta.Chr.4.849, Ta.Chr4.850) which are a part of protein modification and involved in DNA repair [45]. Another gene encodes E3 ubiquitin protein ligase RIE1 (Ta.Chr.7.3130), which plays an essential role in seed development and embryo development ending in seed dormancy [46]. Moreover, one candidate gene encoded protein DELAY OF GERMINATION 1 (DOG1)-like 1 (Ta.Chr.2.2339), which is involved in controlling seed dormancy, e.g., *DOG1* expression is associated with seed maturation temperature effects on germination [47]. DOG1 was also previously identified as a PSG in a genome-wide variation study in worldwide *A. thaliana* accessions and has a major role in increasing seed dormancy in accessions collected from low latitudes [48]. Other PSGs in HG include the transcription factor MYB 27, MYB 98, and abscisic acid receptor PYL13. These candidate PSGs may represent the genetic complexes adapted to extreme environmental conditions on the QTP.

### A single base mutation causes early flowering in high-elevation populations

**Fig. 4.**
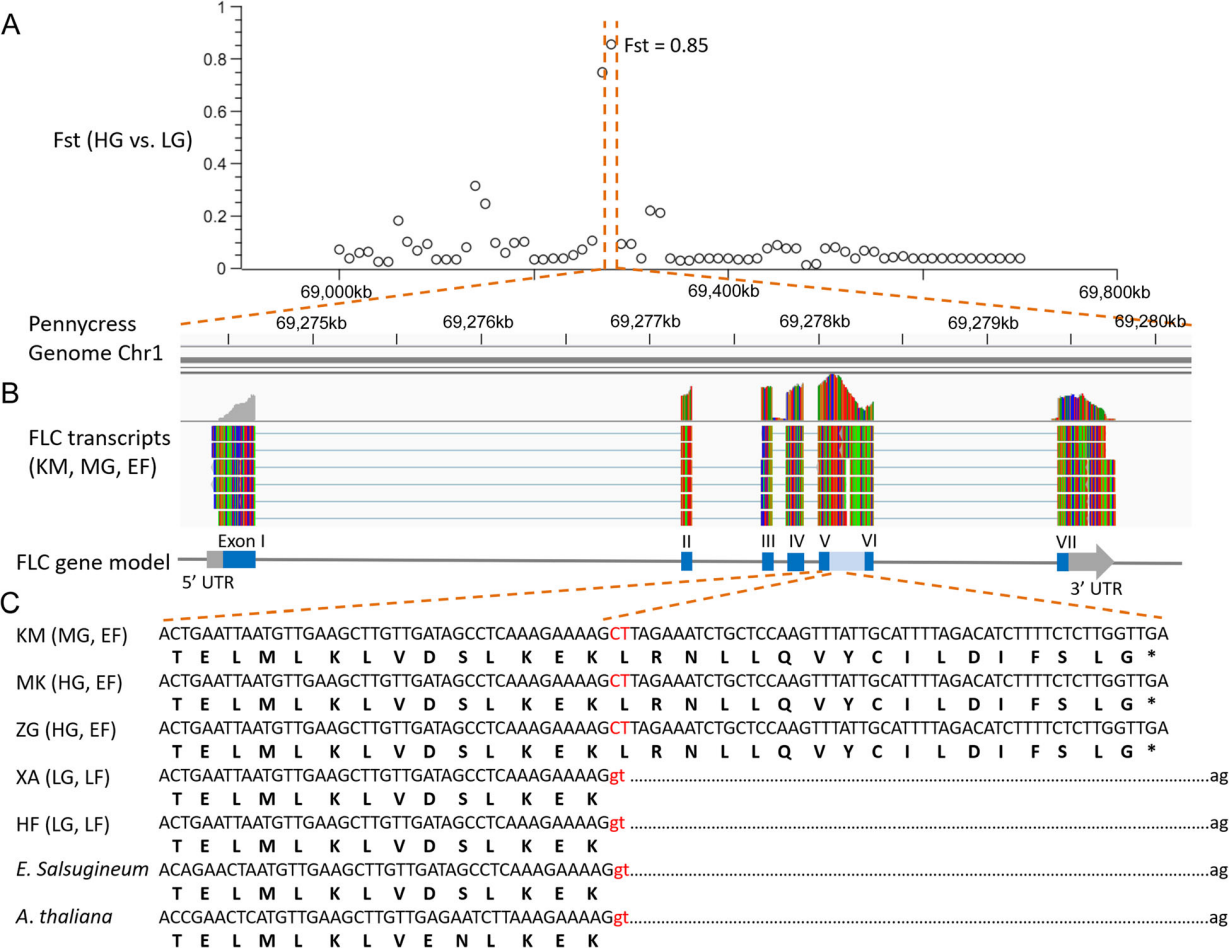
Transitioning to early flowering in the high-elevation groups. **a** The Fst values between HG and LG in the genome region (Chr1:69,000–69,800 kb) which includes the *FLC* gene. **b** The *FLC* transcripts in the Kunming (KM) accession and corresponding gene model. The former fifth intron was transcribed (pale blue) and resulted in a new long transcript formed from the fifth to the sixth exons (blue). **c** Nucleic acid and amino acid sequence alignments of the fifth exon and partial fifth intron of the *FLC* gene from pennycress as well as *A. thaliana* and *E*. *salsugineum*. The mutations from “GT” to “CT” in the fifth intron are highlighted in red. HG, high-elevation group; MG, middle-elevation group; LG, low-elevation group; EF, early flowering; LF, late flowering

Our laboratory experiment showed that the flowering time of field pennycress from the QTP is 18–29 days, significantly less than accessions from low-elevation populations (XA) where flowering time is about 5 months (140–156 days) [20]. Previous studies suggested that vernalization increased the rate of flowering in the late flowering pennycress accessions [20, 49]. In the vernalization pathway, FLOWERING LOCUS C (FLC) is a key repressor of flowering in the late-flowering phenotype, which could be induced by the FRIGIDA (FRI)-complex and in turn repress the floral integrators FLOWERING LOCUS T (FT) [50]. To detect candidate genes that might explain the early-flowering phenotype in high-elevation populations, we mapped re-sequenced short reads of HG and LG to the field pennycress genome. We did not detect any fixed mutations in *FRI*, *FT*, and other flowering-related genes (e.g., *CONSTANS*, *GIGANTEA*, *CDF*, *SPL*, *PIF*, and *TSF*) between HG and LG accessions (Additional file 18: Figure S4). Interestingly, we found that *FLC* was not annotated in our reference genome of the Kunming accession (alt. 1910 m, early flowering). To investigate whether *FLC* (pseudo-)gene does exist in our reference genome, we amplified the sequence using five paired primers (Additional file 19: Figure S5) and then located its position in the reference genome: Chr1:69,274,372–69,279,908. We also mapped the short reads of the four populations to this genome region, and a “G” to “C” (c.450+1 G>C) mutation was observed between all high- and low-elevation accessions (Additional file 20: Figure S6). Moreover, the F_ST_ value (0.85) between HG and LG in this genome region which includes the *FLC* gene is very high compared to the mean F_ST_ value (0.1818), suggesting that this gene locus has undergone strong natural selection in high-elevation environments (Fig. 4a).

Notably, this mutation is in the starting position (GT>CT) of splicing recognition in the fifth intron and might cause abnormal intron splicing. This speculation was confirmed by our transcriptome results of the Kunming accession, which showed the former fifth intron was transcribed and a new long transcript was formed from the fifth to the sixth exons (Fig. 4b). However, a stop codon (TGA) appears after 51 bases from the mutation site “C” (Fig. 4c), which results in an incomplete K-box domain due to the absence of the sixth and seventh exons. Therefore, all of these results suggest that this loss-of-function mutation of the FLC protein might account for early flowering traits in the high-elevation group [50]. As alpine plants living on the QTP usually experience a very short vegetation growing season, flowering time is particularly critical and affects both the life cycles and reproductive success of alpine plants [51, 52]. A single base mutation in *FLC* detected in high-altitude populations of field pennycress may facilitate rapid adaptation to the shorter growing season on the QTP. These findings in combination with other studies suggest that the flowering-time variation of Brassicaceae species has arisen on many independent occasions [17, 53].

## Conclusion

The present study is of scientific importance as it describes how an emerging model plant has evolved and adapted to extreme environmental conditions. Field pennycress is a valuable oilseed crop and lives in diverse environments from sea level to nearly 4500 m high on the QTP. We first de novo sequenced and assembled a high-quality chromosome-level field pennycress genome. Then, we studied the evolutionary genomics and genome-wide polymorphisms associated with altitude variation in different populations of field pennycress. Our study has identified several physiological processes and related genes implicated for adaptation to environments with extreme elevation differences. We also detected a single base mutation causing loss-of-function of the FLC protein, and which is the reason for the transition to early flowering in high-elevation populations of field pennycress. Although further experimental verification is needed, our study provides a genome-wide perspective of how plants adapt to harsh and distinct environments and differs from previous studies focusing on hypoxia adaptation in animals on the QTP [8].

## Methods

### Plant materials, genome sequencing, and assembly

Seedlings of field pennycress were sampled from Kunming (KM, alt. 1910 m, N 30.313°, E 99.358°), southwest of China. Seedlings from the same individual were cultivated in the greenhouse at Yunnan University. The field pennycress genome sequencing and assembly were performed using reads obtained from a combination of sequencing technologies: Illumina Hiseq and Oxford Nanopore (Table S1). First, paired-end libraries were prepared, and short reads (~ 150 bp) generated using Illumina HiSeq platforms. Genome size and heterozygosity of pennycress were estimated using *k*-mer statistics [22] based on the HiSeq reads. Next, the Nanopore library (30–80 kb) was sequenced on R9.4 flow cells using the PromethION DNA sequencer (Oxford Nanopore Technologies, NY, USA). For the Nanopore data, reads with mean quality scores > 7 were retained and then assembled into contigs by the program wtdbg ver.2.4 [54]. Then, GapCloser from the SOAPdenovo2 package [55] was used for gap filling within assembled contigs using pair-end short reads. Lastly, SSPACE [56] was used to improve the assembled draft genome.

### Chromosome assignment using Hi-C

For Hi-C sequencing, the library preparation procedure was conducted as previously described [57]. The libraries were controlled and sequenced on the Illumina HiSeq platform (paired-end sequencing with 150 bp length). The Hi-C paired-end clean reads were aligned to the assembled contigs with BWA-mem (v. 0.7.17) [58] and then clustered onto chromosomes with LACHESIS (http://shendurelab.github.io/LACHESIS/). The whole steps of library construction and sequencing of Illumina, Nanopore, and Hi-C were performed at Novogene Bioinformatics Technology Co., Ltd (Beijing, China).

### Gene prediction and annotation

Gene prediction was performed using a combination of homology, de novo, and transcriptome-based approaches. Total RNA was extracted from the leaf, flower, and root tissues of the same individuals’ offspring for genome sequencing using the QIAGEN RNeasy Plant Mini Kit (QIAGEN, Hilden, Germany). RNA-seq libraries were then prepared using the TruSeq RNA Library Preparation Kit (Illumina, CA, USA), and paired-end sequencing with a read length of 150 bp was conducted on the Illumina HiSeq platform.

Gene models were integrated by EvidenceModeler (http://evidencemodeler.sourceforge.net). Gene models were further updated by PASA [59] to generate UTRs and provide information on alternative splicing variants. The predicted genes were analyzed for functional domains and homologs using InterProScan and BLAST against the NCBI non-redundant protein sequence database, TrEMBL, and SwissProt with an E-value cutoff of 1e−15 and Blast2GO with default parameters. Completeness of the genome was also assessed by performing core gene annotation using the Benchmarking Universal Single Copy Orthologs (BUSCO) methods [23]. Transcription factors were identified and classified into different families using the iTAK pipeline (http://bioinfo.bti.cornell.edu/cgi-bin/itak/index.cgi) [60].

### Whole-genome alignment and repetitive elements analysis

We aligned the field pennycress genome to *Brassica rapa* using LASTZ [61]. In order to avoid the interference caused by repetitive sequences for sequence alignment, RepeatMasker and RepBase library (version 18.08) were used to mask repetitive sequences of both genomes. The raw alignments were combined into larger blocks using the ChainNet algorithm. MCscanx [62] was used to identify syntenic blocks within the genome. For each gene pair in a syntenic block, the 4DTv (transversion substitutions at fourfold degenerate sites) distance was calculated; values of all gene pairs were plotted to identify putative whole-genome duplication events and divergence in two species.

We examined de novo predicted repetitive sequences using RepeatProteinMask and RepeatMasker (http://www.repeatmasker.org) with the Repbase library (version 18.08) [63] and Tandem repeats finder (TRF) [64] (http://tandem.bu.edu/trf/trf.unix.help.html). Prediction of transposable elements (TEs) of field pennycress used a combined strategy based on homologous sequence alignment and de novo searches. De novo TE prediction was performed using RepeatModeler (http://www.repeatmasker.org/RepeatModeler.html), RepeatScout (http://www.repeatmasker.org), Piler [65] (http://www.drive5.com/piler/), LTRharvest [66], and LTR-Finder [67] (http://tlife.fudan.edu.cn/ltr_finder) with default parameters. For the alignment of homologous sequences to identify repeats in the assembled genome, we used RepeatProteinMask and RepeatMasker (http://www.repeatmasker.org) with the RepBase library (version 18.08) [63]. Transposable elements overlapping with the same type of repeats were integrated, while those with low scores were removed if they overlapped over 80% of their lengths and belonged to different types. The repeats that could not be sorted by Repbase were classified as unknown. The LTR-RTs were identified using LTR harvest with options (-similar 85 -vic 10 -seed 20 -seqids yes -minlenltr 100 -maxlenltr 7000 -mintsd 4 -maxtsd 6) and LTR Finder 1.0.6 with options (-w 2 -D 15000 -d 1000 -L 7000 -l 100 -p 20 -C -M 0.85). The identified LTRs from these tools were later integrated by running LTR-RETRIEVER 2.8.4 [68] to evaluate the accuracy and completeness. LTR 5′ and 3′ pairs were aligned in MUSCLE, and the genetic distance between LTR pairs was calculated using the Jukes-Cantor (JC69) nucleotide substitution models. Insertion times were converted into million years using a substitution rate (r) of 7 × 10^−9^ substitutions per site per year [69, 70], and the insertion date (T) was calculated for each LTR retrotransposons (T = K/2r, K: genetic distance).

### Phylogenetic tree construction and divergence time estimation

We selected genomes of field pennycress and eleven other species (*A. thaliana*, *A. lyrata*, *Capsella rubella*, *Cardamine hirsuta*, *Eutrema salsugineum*, *Brassica rapa*, *Brassica oleracea*, *Schrenkiella parvula*, *Arabis alpina*, *Aethionema arabicum*, and *Tarenaya hassleriana*) to identify orthologs. Protein sequences of these plants were compared with each other using BLASTP (E value < 1e−10) and clustered into orthologous groups using OrthoFinder2 [31]. MUSCLE [71] was used to generate multiple sequence alignment for protein sequences in each single-copy group with default parameters. The alignments of each family were concatenated to a super alignment matrix, which was then used for phylogenetic tree reconstruction through the JTT+F+R3 model in IQ-TREE [72] with 1000 bootstraps.

The divergence time between all species was estimated using MCMCtree in PAML [34] with the options “independent rates” and “GTR” model. A Markov chain Monte Carlo analysis was run for 10,000 generations, using a burn-in of 1000 iterations. Three calibration points were applied based on a previous study of Brassicales: *Aethionema arabicum* and other crucifers divergence time (29.0–41.8 Mya), core Brassicaceae origination time (21.3–29.8 Mya), and core *Arabidopsis* origination time (4.8–9.7 Mya) [35].

### Population resequencing and SNP calling

Field pennycress seedlings were collected from two high-elevation areas: Mangkang (MK, 3751 m elevation) and Zuogong (ZG, 4045 m elevation) in Tibet, and two low-elevation areas: Xi’an (XA, 766 m elevation) and Hefei (HF, 31 m elevation). Seedlings of ten individuals were collected in each area. A total of 40 seedlings were cultivated in a greenhouse at Yunnan University. Genomes were re-sequenced by standard procedures on the Illumina HiSeq X Ten platform to yield 150 bp paired-end reads with an insert size around 300 bp. To ensure that reads were reliable and without artificial bias, raw data was firstly processed through a series of quality control (QC) procedures. QC standards were as follows: (1) removing reads with ≥ 10% unidentified nucleotides (N); (2) removing reads with > 50% bases having a phred quality < 5; (3) removing reads with > 10 nt aligned to the adapter, allowing ≤ 10% mismatches; and (4) removing putative PCR duplicates generated in the library construction process.

After trimming low-quality bases, paired-end reads of each population were mapped to our field pennycress reference genome using BWA-mem (v. 0.7.17) [58] with parameters of mem -t 4 -k 32 -M -R. Ambiguously mapped reads were removed. Alignment files were converted to BAM files using the SAMtools v0.1.18 software [73]. Variant calling was performed for all samples using the UnifiedGenotyper function in GATK software [74]. SNPs used the VariantFiltration parameter in GATK (settings: --filterExpression “QD < 4.0 || FS > 60.0 || MQ < 40.0”, -G_filter “GQ<20”, --clusterWindowSize 4). ANNOVAR [75] was used to annotate SNPs based on the GFF3 files for our field pennycress reference genome.

### Genetic diversity and population structure

We studied genetic variation within populations by calculating nucleotide diversity θ_π_, Watterson’s θ, and Tajima’s D separately for each population with ANGSD [76]. Genetic structure based on SNP variation was analyzed using ADMIXTURE [38] with 2–15 ancestral clusters (K), and the value K = 2 was selected using the chooseK.py function. A neighbor-joining tree was constructed using MEGA [77] with the p-distance method, and the clade supports were calculated using 1000 bootstraps. Principal component analysis (PCA) was performed with GCTA [78].

The population genetic differentiation, Fst, was calculated using a method described previously [79, 80] with a window size of 20 kb with 50% overlapping step size. In order to avoid bias and reduce false positives of genetic differentiation between the two high- and two low-elevation populations, we calculated the root mean square (rms) of F_ST_ according to the following formula [81]:

$$ \mathrm{Fst}\left(\mathrm{rms}\right)=\sqrt{1/4\Big(\left(\mathrm{Fst}{\left({\mathrm{H}}_1,{\mathrm{L}}_1\right)}^2+\mathrm{Fst}{\left({\mathrm{H}}_1,{\mathrm{L}}_2\right)}^2+\mathrm{Fst}{\left({\mathrm{H}}_2,{\mathrm{L}}_1\right)}^2+\mathrm{Fst}{\left({\mathrm{H}}_2,{\mathrm{L}}_2\right)}^2\right)} $$.

We also used the average expected nucleotide heterozygosity within a population (θ_π_) to detect selected loci of each population. To identify regions that were likely to be or have been under selection, the F_ST_(rms) and θ_π_ combined approach was used, as previously described [43, 44]. Furthermore, the selective sweeps were also detected using a composite likelihood ratio (CLR) based on the Sweepfinder [82]. For candidate-selected genes in population genomics analysis between high- and low-elevation populations, the Gene Ontology (GO) enrichment analyses were performed using the R package ClusterProfiler [83] to identify significantly enriched terms. The KOBAS and BlastKOALA software [84] were also used to test the statistical enrichment of genes in Kyoto Encyclopedia of Genes and Genomes (KEGG) pathways [85]. The resulting *P* values were corrected for multiple comparisons using the method of Benjamini and Hochberg.

### LD analysis and demographic estimation

Linkage disequilibrium (LD) based on the coefficient of determination (r^2^) was calculated between each pair of SNPs using PopLDdecay (https://github.com/BGI-shenzhen/PopLDdecay). Recent demographic history was measured by the trend in effective population size (Ne) change over time using Stairway Plot [39]. The program uses the unfolded site frequency spectrum (SFS) to infer population size changes over time. We used ANGSD first to generate the sample allele frequency (SAF) for each population, and then used realSFS to generate the population SFS. Loci with missing data were excluded from this analysis, as well as SNPs with a minimum allele frequency (MAF) smaller than 5%. The estimated generation time (g) was set as 1 year, and the mutation rate was 7 × 10^−9^ mutations per generation per site.

### FLOWERING LOCUS C (FLC) variation and early flowering in high-elevation populations

The *FLC* gene has not been annotated in our reference genome of the Kunming accession. To make sure the reference genome has this (pseudo-) gene sequence, we firstly designed five paired PCR primers according to the known *FLC* sequence of a North America accession (Additional file 19: Figure S5) [86]. Then, the amplified gene sequence of the Kunming accession was used as a query to search the reference genome and identify its position (Chr1:69,274,372–69,279,908). Using the standard BWA-mem [58] and Samtools (v. 1.6) [73] pipeline, we mapped re-sequenced short reads of four populations to the reference genome and then manually explored the coverage of the *FLC* region using IGV (v. 2.4.13) [87]. The partial sequence of the predicted *FLC* gene in five areas of China, together with sequences from *A. thaliana* and *Eutrema salsugineum*, was added to the curated alignment using MUSCLE (v. 3.8.31) [71]. We also used transcriptome data to detect *FLC* transcription in Kunming accessions.

## Supplementary Information

**Additional file 1: Figure S1.** Frequency distribution of depth of *K-*mer = 17 in genome survey of field pennycress.

**Additional file 2: Table S1.** Statistics of characteristics of field pennycress genome (K-mer = 17).

**Additional file 3: Table S2.** Statistic of chromosomes of field pennycress using Hi-C technology

**Additional file 4: Table S3.** Information of gene prediction in field pennycress genes.

**Additional file 5: Table S4.** Information of function annotation of field pennycress genes.

**Additional file 6: Table S5.** Statistics of predicted protein-coding genes in field pennycress and relatives.

**Additional file 7: Table S6.** Scaffolds from the field pennycress assembly were aligned to conserved genes using BUSCO method.

**Additional file 8: Figure S2.** Syntenic dot plot and Ks distribution between *T. arvense* and two closely related species; above: *Eutrema salsugineum* (1:1), below: *Brassica oleracea* (1:3).

**Additional file 9: Table S7.** Statistics of repeat sequences (above) and transposable elements (TEs, below) in field pennycress genome.

**Additional file 10: Table S8.** Percentages of transposable elements in reported genomes of Brassicaceae.

**Additional file 11: Table S9.** Data quality overview for all re-sequenced samples.

**Additional file 12: Table S10.** Pairwise F_ST_ value between populations.

**Additional file 13: Figure S3.** Linkage disequilibrium (LD) patterns for the two distinct altitude groups of field pennycress.

**Additional file 14: Table S11.** List of 359 candidate positively selected genes in HG of field pennycress based on top 3% cutoff of both F_ST_(rms) and θπ ratio.

**Additional file 15: Table S12.** GO and KEGG functional categories of 359 candidate positively selected genes in HG based on top 3% cutoff of both F_ST_(rms) and θπ ratio.

**Additional file 16: Table S13.** GO and KEGG functional categories of 621 candidate positively selected genes in HG based on the CLR test.

**Additional file 17: Table S14.** List of 130 positively selected genes in HG based on three selection tests [FST(rms), θπ ratio and CLR].

**Additional file 18: Figure S4**. Mapping re-sequenced short reads of four populations to the reference genome and the coverage of the flowering related genes (*FRI*, *FT*, *CONSTANS*, *GIGANTEA*, *CDF*, *SPL*, *PIF* and *TSF*) region using IGV.

**Additional file 19: Figure S5.** Design of five paired PCR amplification primers for *FLC* gene.

**Additional file 20: Figure S6.** Mapping re-sequenced short reads of four populations to the reference genome and the coverage of the *FLC* region using IGV. The red dotted box shows the mutation site (G>C).

## Data Availability

All data generated or analyzed during this study are included in this published article, its supplementary information files, and publicly available repositories. All genome data generated during this study are deposited in the National Center for Biotechnology Information (NCBI) as BioProject PRJNA715950. The GI number of the *FLC* gene of Kunming in the NCBI is MW716251.
