# Supplementary figures and images for: Genomic analysis of field pennycress (Thlaspi arvense) provides insights into mechanisms of adaptation to high elevation
Source: BMC Biol. 2021 Jul 22;19:143. doi: 10.1186/s12915-021-01079-0 (PMC8296595; doi:10.1186/s12915-021-01079-0)

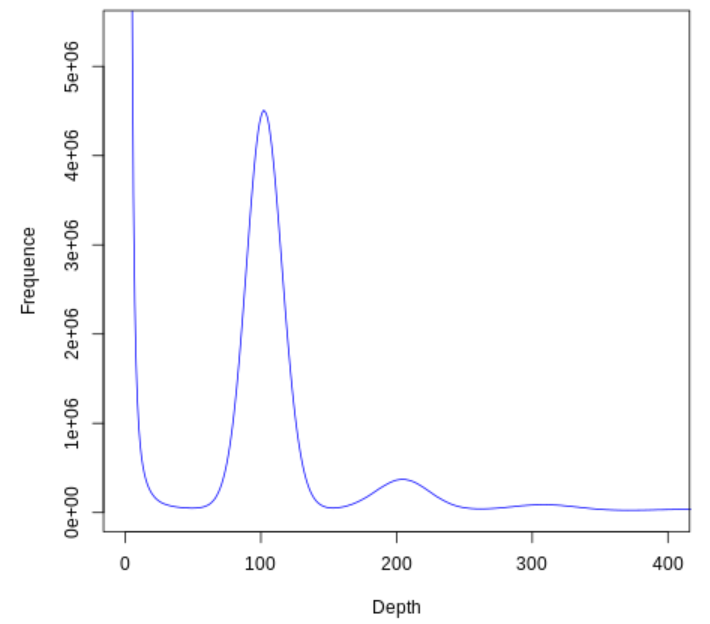


Figure S1. Frequency distribution of depth of *K-mer* = 17 in genome survey of field pennycress.

Supplement: Supplementary file 1 — Additional file 1: Figure S1. Frequency distribution of depth of K-mer = 17 in genome survey of field pennycress. [file 12915_2021_1079_MOESM1_ESM.docx]

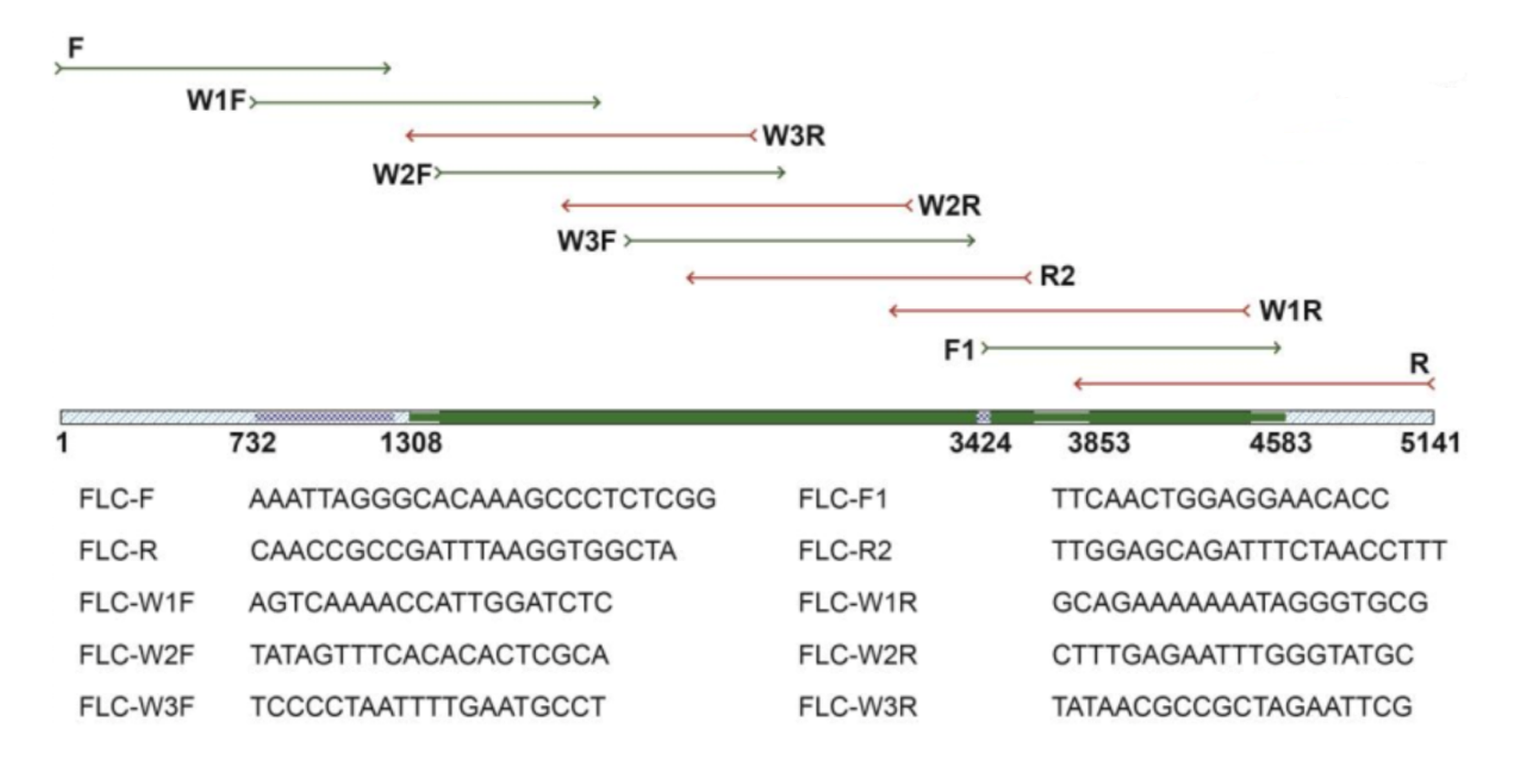


Figure S5. Design of five paired PCR amplification primers for *FLC* gene.

Supplement: Supplementary file 19 — Additional file 19: Figure S5. Design of five paired PCR amplification primers for FLC gene. [file 12915_2021_1079_MOESM19_ESM.docx]
