# Supplementary material for: Genomic analysis of field pennycress (Thlaspi arvense) provides insights into mechanisms of adaptation to high elevation
Source: BMC Biol. 2021 Jul 22;19:143. doi: 10.1186/s12915-021-01079-0 (PMC8296595; doi:10.1186/s12915-021-01079-0)
Supplement: Supplementary file 2 — Additional file 2: Table S1. Statistics of characteristics of field pennycress genome (K-mer = 17). [file 12915_2021_1079_MOESM2_ESM.docx]

**Table S1. Statistics of characteristics of field pennycress genome (*K*-mer = 17)**

| *K*-mer | Depth | *K*-mer Num. | Genome size | Revised Genome size | Heterozygous rate | Repeat rate |
| --- | --- | --- | --- | --- | --- | --- |
| 17 | 103 | 56,941,495,305 | 552.83Mb | 548.21Mb | 0.07% | 69.47% |
