# Supplementary material for: Genomic analysis of field pennycress (Thlaspi arvense) provides insights into mechanisms of adaptation to high elevation
Source: BMC Biol. 2021 Jul 22;19:143. doi: 10.1186/s12915-021-01079-0 (PMC8296595; doi:10.1186/s12915-021-01079-0)
Supplement: Supplementary file 3 — Additional file 3: Table S2. Statistic of chromosomes of field pennycress using Hi-C technology [file 12915_2021_1079_MOESM3_ESM.docx]

**Table S2. Statistic of chromosomes of field pennycress using Hi-C technology**

| Chromosome id. | Length (bp) |
| --- | --- |
| Chr1 | 75,835,427 |
| Chr2 | 65,400,620 |
| Chr3 | 57,500,524 |
| Chr4 | 70,797,862 |
| Chr5 | 73,311,318 |
| Chr6 | 59,680,398 |
| Chr7 | 72,450,090 |
| Total | 474,976,239 (90.1%) |
