# Supplementary material for: Genomic analysis of field pennycress (Thlaspi arvense) provides insights into mechanisms of adaptation to high elevation
Source: BMC Biol. 2021 Jul 22;19:143. doi: 10.1186/s12915-021-01079-0 (PMC8296595; doi:10.1186/s12915-021-01079-0)
Supplement: Supplementary file 4 — Additional file 4: Table S3. Information of gene prediction in field pennycress genes. [file 12915_2021_1079_MOESM4_ESM.docx]

**Table S3. Information of gene prediction in field pennycress genes.**

| **Methods** | **Gene set** | **Number** | **Average transcript length(bp)** | **Average CDS length(bp)** | **Average exons per gene** | **Average exon length(bp)** | **Average intron length(bp)** |
| --- | --- | --- | --- | --- | --- | --- | --- |
| De novo | Augustus | 33,465 | 1,664.19 | 1,014.06 | 4.34 | 233.66 | 194.65 |
|  | GlimmerHMM | 59,512 | 1,130.67 | 646.29 | 2.60 | 248.16 | 301.92 |
|  | SNAP | 31,096 | 3,986.46 | 587.24 | 3.68 | 159.63 | 1,268.92 |
|  | Geneid | 42,686 | 2,883.13 | 868.29 | 4.16 | 208.83 | 638.04 |
|  | Genscan | 27,926 | 8,702.07 | 1,313.46 | 6.25 | 210.10 | 1,406.96 |
| Homolog | Aly | 23,254 | 1,897.70 | 1,092.32 | 4.79 | 227.81 | 212.23 |
|  | Aal | 22,408 | 1,924.98 | 1,099.37 | 4.83 | 227.71 | 215.67 |
|  | Bra | 22,327 | 2,016.00 | 1,110.36 | 4.78 | 232.10 | 239.33 |
|  | Bol | 23,824 | 1,915.15 | 1,055.36 | 4.69 | 225.07 | 233.06 |
|  | Ath | 22,863 | 1,874.48 | 1,124.11 | 4.88 | 230.42 | 193.47 |
|  | Bna | 24,120 | 1,834.03 | 1,024.05 | 4.64 | 220.65 | 222.46 |
| RNAseq | PASA | 49,569 | 1,794.18 | 1,011.32 | 4.91 | 205.83 | 200.05 |
|  | Transcripts | 22,883 | 3,578.44 | 1,975.90 | 6.60 | 299.17 | 285.93 |
| EVM | | 39,061 | 1,696.84 | 939.00 | 4.09 | 229.49 | 245.12 |
| Pasa-update | | 38,845 | 1,697.33 | 950.34 | 4.11 | 231.14 | 240.07 |
| Final set | | 31,596 | 1,914.13 | 1,065.09 | 4.59 | 232.26 | 236.79 |
