# Supplementary material for: Genomic analysis of field pennycress (Thlaspi arvense) provides insights into mechanisms of adaptation to high elevation
Source: BMC Biol. 2021 Jul 22;19:143. doi: 10.1186/s12915-021-01079-0 (PMC8296595; doi:10.1186/s12915-021-01079-0)
Supplement: Supplementary file 5 — Additional file 5: Table S4. Information of function annotation of field pennycress genes. [file 12915_2021_1079_MOESM5_ESM.docx]

**Table S4. Information of function annotation of field pennycress genes.**

| #Database | | Annotated Number | Annotated Percent (%) |
| --- | --- | --- | --- |
| NR | | 30,896 | 97.80 |
| Swiss-Prot | | 22,834 | 72.30 |
| KEGG | | 23,126 | 73.20 |
| InterPro | All | 28,025 | 88.70 |
|  | Pfam | 22,821 | 72.20 |
|  | GO | 15,633 | 49.50 |
| Annotated | | 31,026 | 98.20 |
| Total | | 31,596 | - |
