# Supplementary material for: Genomic analysis of field pennycress (Thlaspi arvense) provides insights into mechanisms of adaptation to high elevation
Source: BMC Biol. 2021 Jul 22;19:143. doi: 10.1186/s12915-021-01079-0 (PMC8296595; doi:10.1186/s12915-021-01079-0)
Supplement: Supplementary file 6 — Additional file 6: Table S5. Statistics of predicted protein-coding genes in field pennycress and relatives. [file 12915_2021_1079_MOESM6_ESM.docx]

**Table S5. Statistics of predicted protein-coding genes in field pennycress and relatives**

| Species | Number | Average gene length (bp) | Average CDS length (bp) | Average exons per gene | Average exon length (bp) | Average intron length (bp) |
| --- | --- | --- | --- | --- | --- | --- |
| *T. Thlaspi* | 31,596 | 1,914.13 | 1,065.09 | 4.59 | 232.26 | 236.79 |
| *B. napus* | 101,040 | 1,764.75 | 1,001.16 | 4.91 | 204.06 | 195.48 |
| *B. rapa* | 46,250 | 1,859.41 | 1,125.79 | 4.79 | 234.8 | 193.34 |
| *A. thaliana* | 48,359 | 2,056.72 | 1,296.77 | 5.92 | 219 | 154.42 |
| *B. oleracea* | 59,220 | 1,749.70 | 1,042.26 | 4.54 | 229.8 | 200.1 |
| *A. lyrata* | 33,132 | 1,862.74 | 1,165.52 | 5.11 | 227.98 | 169.54 |
| *Arabis alpina* | 34,220 | 2,444.22 | 1,040.03 | 4.7 | 221.15 | 379.23 |
