# Supplementary material for: Genomic analysis of field pennycress (Thlaspi arvense) provides insights into mechanisms of adaptation to high elevation
Source: BMC Biol. 2021 Jul 22;19:143. doi: 10.1186/s12915-021-01079-0 (PMC8296595; doi:10.1186/s12915-021-01079-0)
Supplement: Supplementary file 7 — Additional file 7: Table S6. Scaffolds from the field pennycress assembly were aligned to conserved genes using BUSCO method. [file 12915_2021_1079_MOESM7_ESM.docx]

**Table S6. Scaffolds from the field pennycress assembly were aligned to conserved genes using BUSCO method.**

| Species | Genome Size | BUSCO annotation assessment results |
| --- | --- | --- |
| **Field pennycress** | 527.15Mb | C: 95.9% [D:16%], F:1.4%, M:2.1%, n:1440 |

C: Complete Single-Copy BUSCOs

D: Complete Duplicated BUSCOs

F: Fragmented BUSCOs

M: Missing BUSCOs

n: Total BUSCO groups searched
