# Supplementary material for: Genomic analysis of field pennycress (Thlaspi arvense) provides insights into mechanisms of adaptation to high elevation
Source: BMC Biol. 2021 Jul 22;19:143. doi: 10.1186/s12915-021-01079-0 (PMC8296595; doi:10.1186/s12915-021-01079-0)
Supplement: Supplementary file 8 — Additional file 8: Figure S2. Syntenic dot plot and Ks distribution between T. arvense and two closely related species; above: Eutrema salsugineum (1:1), below: Brassica oleracea (1:3). [file 12915_2021_1079_MOESM8_ESM.docx]

| 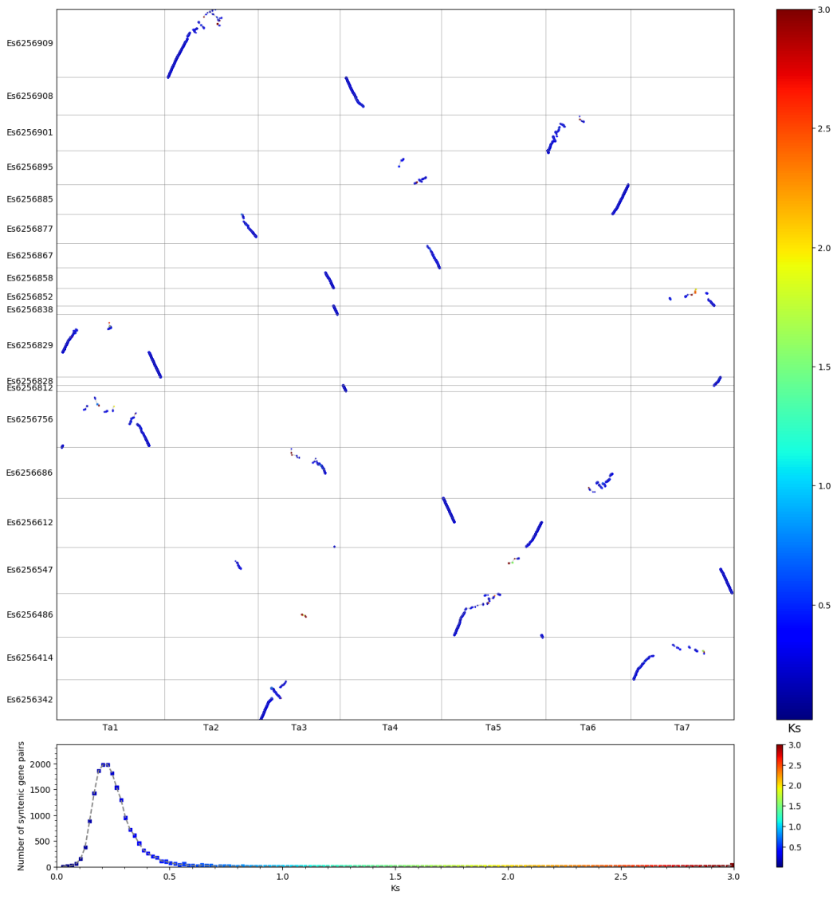  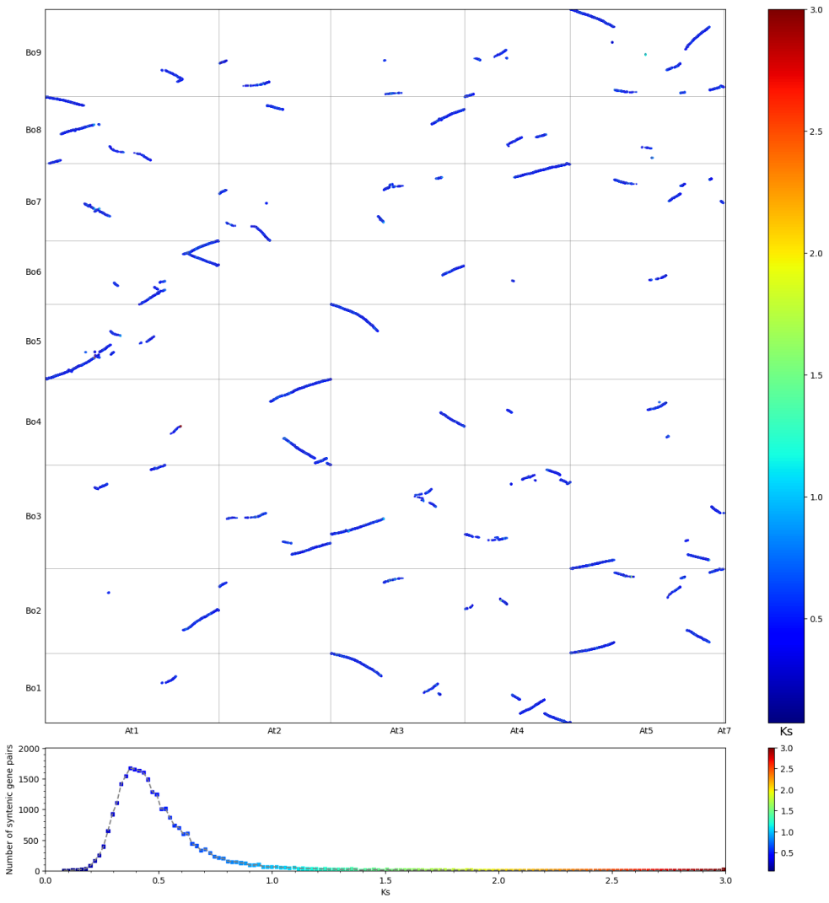 |
| --- |

Figure S2. Syntenic dot plot and Ks distribution between *T. arvense* and two closely related species; above: *Eutrema salsugineum* (1:1), below: *Brassica oleracea* (1:3).
