# Supplementary material for: Genomic analysis of field pennycress (Thlaspi arvense) provides insights into mechanisms of adaptation to high elevation
Source: BMC Biol. 2021 Jul 22;19:143. doi: 10.1186/s12915-021-01079-0 (PMC8296595; doi:10.1186/s12915-021-01079-0)
Supplement: Supplementary file 9 — Additional file 9: Table S7. Statistics of repeat sequences (above) and transposable elements (TEs, below) in field pennycress genome. [file 12915_2021_1079_MOESM9_ESM.docx]

**Table S7. Statistics of repeat sequences (above) and transposable elements (TEs, below) in field pennycress genome.**

| Type | | Repeat Size | | percent of genome (%) | |  |  |  |  |  |
| --- | --- | --- | --- | --- | --- | --- | --- | --- | --- | --- |
| Trf | | 34,579,430 | | 6.56 | |  |  |  |  |  |
| Repeatmasker | | 361,792,297 | | 68.61 | |  |  |  |  |  |
| Proteinmask | | 110,364,798 | | 20.93 | |  |  |  |  |  |
| Total | | 374,753,376 | | 71.07 | |  |  |  |  |  |
| Type | | Denovo+Repbase | | | | TE proteins | | | Combined TEs | |
|  |  | Length (Bp) | | percent of genome (%) | | Length (Bp) | | percent of genome (%) | Length (Bp) | percent of genome (%) |
| ClassII | |  | |  | |  | |  |  |  |
| DNA | | 12,304,464 | | 2.33 | | 2,039,370 | | 0.39 | 13,806,021 | 2.62 |
| ClassI | |  | |  | |  | |  |  |  |
| LINE | | 3,165,421 | | 0.60 | | 4,244,856 | | 0.81 | 6,792,104 | 1.29 |
| SINE | | 5,780,840 | | 1.10 | | 0 | | 0 | 5,780,840 | 1.10 |
| LTR | | 322,570,230 | | 61.17 | | 104,082,652 | | 19.74 | 327,601,384 | 62.13 |
| Unknown | | 26,597,650 | | 5.04 | | 0 | | 0 | 26,597,650 | 5.04 |
| Total | | 361,792,297 | | 68.61 | | 110,364,798 | | 20.93 | 370,130,998 | 70.19 |

| Subtype of Class II | Length | Percent |
| --- | --- | --- |
| DNA/Academ | 364 | 0.00% |
| DNA/CMC | 2360486 | 0.45% |
| DNA/Crypton | 4213 | 0.00% |
| DNA/CryptonF | 54 | 0.00% |
| DNA/CryptonV | 4246 | 0.00% |
| DNA/DNA | 221145 | 0.04% |
| DNA/Dada | 1130405 | 0.21% |
| DNA/EnSpm | 132366 | 0.03% |
| DNA/Ginger | 120 | 0.00% |
| DNA/Ginger1 | 2382 | 0.00% |
| DNA/Ginger2 | 652 | 0.00% |
| DNA/Harbinger | 88412 | 0.02% |
| DNA/Helitron | 2585800 | 0.49% |
| DNA/IS3EU | 2230 | 0.00% |
| DNA/ISL2EU | 3186 | 0.00% |
| DNA/Kolobok | 19868 | 0.00% |
| DNA/MULE | 2748210 | 0.52% |
| DNA/Mariner | 10692 | 0.00% |
| DNA/Maverick | 474 | 0.00% |
| DNA/Merlin | 72 | 0.00% |
| DNA/MuDR | 249240 | 0.05% |
| DNA/MuLE | 1024892 | 0.19% |
| DNA/Novosib | 497 | 0.00% |
| DNA/P | 3344 | 0.00% |
| DNA/PIF | 453311 | 0.09% |
| DNA/PiggyBac | 1649736 | 0.31% |
| DNA/Polinton | 11403 | 0.00% |
| DNA/Sola | 7227 | 0.00% |
| DNA/Sola1 | 203 | 0.00% |
| DNA/Sola2 | 49 | 0.00% |
| DNA/Sola3 | 98 | 0.00% |
| DNA/TcMar | 103522 | 0.02% |
| DNA/Transib | 915 | 0.00% |
| DNA/Zisupton | 5667 | 0.00% |
| DNA/hAT | 2117271 | 0.40% |
| DNA/piggyBac | 831 | 0.00% |
