# Supplementary material for: Genomic analysis of field pennycress (Thlaspi arvense) provides insights into mechanisms of adaptation to high elevation
Source: BMC Biol. 2021 Jul 22;19:143. doi: 10.1186/s12915-021-01079-0 (PMC8296595; doi:10.1186/s12915-021-01079-0)
Supplement: Supplementary file 10 — Additional file 10: Table S8. Percentages of transposable elements in reported genomes of Brassicaceae. [file 12915_2021_1079_MOESM10_ESM.docx]

**Table S8. Percentages of transposable elements in reported genomes of** **Brassicaceae.**

| Species | Estimated genome size (Mb) | Assembly size (Mb) | TE (%) | References |
| --- | --- | --- | --- | --- |
| *Pachycladon cheesemanii* | 596 | 422 | 22.8% | Dong et al., BMC Genomics, 2019 |
| *Arabidopsis thaliana* | 160 | 125 | 23.7% | The Arabidopsis Genome Initiative, Nature, 2000;  Hu et al., Nat Genet, 2011;  Long et al., Nat Genet, 2013. |
| *Boechera retrofracta* | 226.87 | 222.25 | 26.08% | Kliver et al., Genes, 2018. |
| *Camelina sativa* | 750 | 641.45 | 28% | Kagale et al., Nat Commun, 2014. |
| *Arabidopsis lyrata* | 230 | 207 | 29.7% | Johnstonet al., Annals of Botany, 2005;  Hu et al., Nat Genet, 2011. |
| *Microthlaspi erraticum* | 211 | 170 | 33.93% | Mishra et al., Frontiers in Plant Science, 2020. |
| *Brassica rapa* | 442.9 | 353.14 | 37.51% | Zhang et al., Horticulture Research, 2018. |
| *Raphanus sativus* | 498.5 | 504.5 | 38.7% | Shirasawa et al., DNA Research, 2020. |
| *Brassica oleracea* | 630 | 539.9 | 38.8% | Liu et al., Nat Commun, 2014. |
| *Crucihimalaya himalaica* | 265.23 | 234.72 | 45.78% | Zhang et al., PNAS, 2019. |
| *Lepidium meyenii* | 751 | 743 | 47.65% | Zhang et al., Mol Plants, 2016. |
| *Arabis alpine* | 375 | 309 | 47.9% | Willing et al., Nature Plants, 2015. |
| *Brassica napus* | 1132 | 924 | 49.78% | Sun et al., The Plant J., 2017 |
| *Capsella rubella* | 219 | 134.8 | 50% | Slotte et al., Nature Genetics, 2013 |
| *Thellungiella salsuginea* | 260 | 233.7 | 52% | Wu et al., PNAS, 2012 |
| *Megacarpaea delavayi* | 899 | 883.81 | 55.76% | Yang et al., Frontiers in Genetics, 2020. |
| *Eutrema heterophyllum* | 405 | 350 | 67% | Guo et al., DNA Research, 2018 |
| *Eutrema yunnanense* | 423 | 413 | 70% | Guo et al., DNA Research, 2018 |
| *Thlaspi arvense* | 548.21 | 527.15 | 70.19% | This study |
