# Supplementary material for: Genomic analysis of field pennycress (Thlaspi arvense) provides insights into mechanisms of adaptation to high elevation
Source: BMC Biol. 2021 Jul 22;19:143. doi: 10.1186/s12915-021-01079-0 (PMC8296595; doi:10.1186/s12915-021-01079-0)
Supplement: Supplementary file 11 — Additional file 11: Table S9. Data quality overview for all re-sequenced samples. [file 12915_2021_1079_MOESM11_ESM.docx]

**Table S9. Data quality overview for all re-sequenced samples.**

| Sample | Raw bases (bp) | Clean bases (bp) | Clean reads number | Q30 (%) | Mapped reads | Mapping rate | Average depth | Coverage > 4X |
| --- | --- | --- | --- | --- | --- | --- | --- | --- |
| HF1 | 9.64E+09 | 9.63E+09 | 64182014 | 91.91 | 63739788 | 99.31% | 18.49 | 82.40% |
| HF10 | 1.02E+10 | 1.02E+10 | 68177802 | 91.92 | 67735701 | 99.35% | 19.25 | 82.43% |
| HF2 | 9.35E+09 | 9.34E+09 | 62271014 | 92.02 | 61841312 | 99.31% | 18.1 | 82.51% |
| HF3 | 9.43E+09 | 9.42E+09 | 62824920 | 91.98 | 62343332 | 99.23% | 18.04 | 82.26% |
| HF4 | 9.77E+09 | 9.77E+09 | 65100814 | 91.97 | 64416413 | 98.95% | 18.81 | 81.82% |
| HF5 | 1.1E+10 | 1.09E+10 | 72991504 | 92.1 | 72511916 | 99.34% | 20.48 | 82.70% |
| HF6 | 9.76E+09 | 9.75E+09 | 64987832 | 91.91 | 64506297 | 99.26% | 18.74 | 82.27% |
| HF7 | 1.02E+10 | 1.02E+10 | 68040734 | 91.94 | 62690704 | 92.14% | 17.89 | 82.05% |
| HF8 | 9.8E+09 | 9.79E+09 | 65263274 | 91.94 | 64830103 | 99.34% | 18.48 | 82.43% |
| HF9 | 1.07E+10 | 1.07E+10 | 71421750 | 92.16 | 70952582 | 99.34% | 19.83 | 82.64% |
| MK1 | 9.57E+09 | 9.55E+09 | 63687842 | 91.42 | 62764542 | 98.55% | 17.8 | 81.22% |
| MK10 | 9.84E+09 | 9.83E+09 | 65506704 | 91.75 | 64724106 | 98.81% | 18.46 | 81.31% |
| MK2 | 9.68E+09 | 9.67E+09 | 64460796 | 91.89 | 63386887 | 98.33% | 18.4 | 81.14% |
| MK3 | 1.01E+10 | 1E+10 | 66940794 | 91.8 | 61555511 | 91.96% | 17.17 | 81.56% |
| MK4 | 9.72E+09 | 9.71E+09 | 64735936 | 92.07 | 63686572 | 98.38% | 18.57 | 81.35% |
| MK5 | 9.9E+09 | 9.89E+09 | 65921944 | 91.78 | 64931862 | 98.50% | 18.88 | 81.09% |
| MK6 | 9.56E+09 | 9.55E+09 | 63674216 | 91.61 | 62722595 | 98.51% | 18.17 | 81.21% |
| MK7 | 9.89E+09 | 9.88E+09 | 65864606 | 92.16 | 65210885 | 99.01% | 18.81 | 81.60% |
| MK8 | 9.44E+09 | 9.42E+09 | 62825578 | 91.98 | 60935741 | 96.99% | 17.95 | 80.65% |
| MK9 | 9.55E+09 | 9.54E+09 | 63572888 | 91.94 | 60935374 | 95.85% | 17.86 | 80.86% |
| XA1 | 1.11E+10 | 1.11E+10 | 73897618 | 92.34 | 73351866 | 99.26% | 21.25 | 82.62% |
| XA10 | 1.1E+10 | 1.1E+10 | 73263036 | 92.56 | 72786554 | 99.35% | 21.29 | 81.93% |
| XA2 | 1.06E+10 | 1.06E+10 | 70476104 | 92.67 | 69941352 | 99.24% | 20.54 | 82.46% |
| XA3 | 1.1E+10 | 1.1E+10 | 73281030 | 92.45 | 72726783 | 99.24% | 21.02 | 82.24% |
| XA4 | 9.02E+09 | 9.01E+09 | 60068534 | 92.19 | 59619371 | 99.25% | 17.45 | 81.64% |
| XA5 | 9.68E+09 | 9.67E+09 | 64446696 | 92.21 | 63985683 | 99.28% | 18.62 | 82.01% |
| XA6 | 1.1E+10 | 1.1E+10 | 73227912 | 92.66 | 72644056 | 99.20% | 21.02 | 81.82% |
| XA7 | 1.09E+10 | 1.09E+10 | 72573608 | 92.51 | 72043911 | 99.27% | 20.8 | 81.94% |
| XA8 | 1.09E+10 | 1.09E+10 | 72410182 | 92.22 | 71788999 | 99.14% | 20.63 | 81.89% |
| XA9 | 9.24E+09 | 9.22E+09 | 61489906 | 92.03 | 60958727 | 99.14% | 17.51 | 81.23% |
| ZG1 | 9.3E+09 | 9.29E+09 | 61927238 | 91.53 | 58836242 | 95.01% | 16.8 | 80.88% |
| ZG10 | 9.1E+09 | 9.09E+09 | 60589208 | 91.72 | 60080821 | 99.16% | 17.51 | 80.78% |
| ZG2 | 1E+10 | 9.99E+09 | 66579586 | 91.75 | 65952803 | 99.06% | 19.48 | 79.36% |
| ZG3 | 1.03E+10 | 1.03E+10 | 68471684 | 91.55 | 67904967 | 99.17% | 19.56 | 81.60% |
| ZG4 | 1.02E+10 | 1.02E+10 | 67678578 | 91.75 | 67159499 | 99.23% | 19.43 | 81.38% |
| ZG5 | 9.95E+09 | 9.93E+09 | 66207046 | 92.12 | 65249366 | 98.55% | 18.72 | 79.25% |
| ZG6 | 1.19E+10 | 1.19E+10 | 79396800 | 92.38 | 78538288 | 98.92% | 22.56 | 81.71% |
| ZG7 | 9.61E+09 | 9.6E+09 | 64004582 | 91.92 | 63528578 | 99.26% | 18.63 | 81.08% |
| ZG8 | 9.56E+09 | 9.55E+09 | 63688302 | 92.32 | 62089307 | 97.49% | 18.27 | 81.43% |
| ZG9 | 1.01E+10 | 1.01E+10 | 67577014 | 91.64 | 66875086 | 98.96% | 19.24 | 81.31% |
