# Supplementary material for: Genomic analysis of field pennycress (Thlaspi arvense) provides insights into mechanisms of adaptation to high elevation
Source: BMC Biol. 2021 Jul 22;19:143. doi: 10.1186/s12915-021-01079-0 (PMC8296595; doi:10.1186/s12915-021-01079-0)
Supplement: Supplementary file 12 — Additional file 12: Table S10. Pairwise FST value between populations. [file 12915_2021_1079_MOESM12_ESM.docx]

**Table S10 Pairwise F_ST_ value between populations.**

| Populations | Fst |
| --- | --- |
| LG vs. HG | 0.1818 |
| XA vs. HF | 0.2056 |
| XA vs. ZG | 0.1872 |
| HF vs. MK | 0.2832 |
| HF vs. ZG | 0.2385 |
| ZG vs. MK | 0.0861 |
| XA vs. MK | 0.2709 |
