# Supplementary material for: Genomic analysis of field pennycress (Thlaspi arvense) provides insights into mechanisms of adaptation to high elevation
Source: BMC Biol. 2021 Jul 22;19:143. doi: 10.1186/s12915-021-01079-0 (PMC8296595; doi:10.1186/s12915-021-01079-0)
Supplement: Supplementary file 13 — Additional file 13: Figure S3. Linkage disequilibrium (LD) patterns for the two distinct altitude groups of field pennycress. [file 12915_2021_1079_MOESM13_ESM.docx]

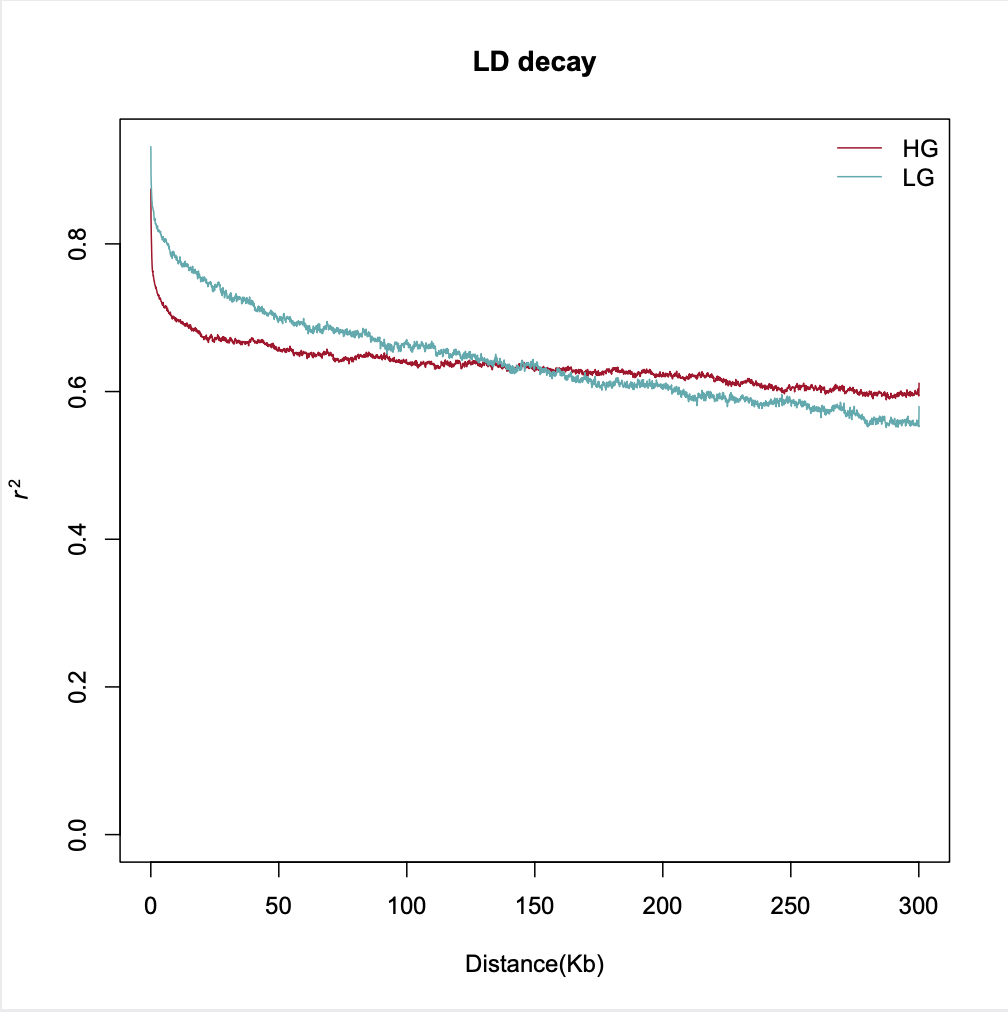


Figure S3 Linkage disequilibrium (LD) patterns for the two distinct altitude groups of field pennycress. X axis: physical distances between two SNPs marked in kb; Y axis: R^2^ used to measure linkage disequilibrium.
