# Supplementary material for: Genomic analysis of field pennycress (Thlaspi arvense) provides insights into mechanisms of adaptation to high elevation
Source: BMC Biol. 2021 Jul 22;19:143. doi: 10.1186/s12915-021-01079-0 (PMC8296595; doi:10.1186/s12915-021-01079-0)
Supplement: Supplementary file 14 — Additional file 14: Table S11. List of 359 candidate positively selected genes in HG of field pennycress based on top 3% cutoff of both FST(rms) and θπ ratio. [file 12915_2021_1079_MOESM14_ESM.docx]

**Table S11. List of 359 candidate positively selected genes in HG of field pennycress based on top 3% cutoff of both FST(rms) and θπ ratio.**

| #Chr | START | END | log2(pi_HG_/pi_LG_) | F_ST_(rms) | PSGs |
| --- | --- | --- | --- | --- | --- |
| Chr1 | 65180000 | 65190000 | -8.793312 | 0.8657416 | Chr1.3980, Chr1.3981, Chr1.3982, Chr1.3983 |
| Chr1 | 67080000 | 67090000 | -4.59647 | 0.7917429 | Chr1.4475, Chr1.4476, Chr1.4477, Chr1.4478 |
| Chr1 | 67085000 | 67095000 | -4.51275 | 0.7985 | Chr1.4477, Chr1.4478, Chr1.4479 |
| Chr1 | 67090000 | 67100000 | -4.428337 | 0.8000152 | Chr1.4478, Chr1.4479, Chr1.4480 |
| Chr1 | 67095000 | 67105000 | -4.468742 | 0.795474 | Chr1.4479, Chr1.4480, Chr1.4481 |
| Chr1 | 67135000 | 67145000 | -7.405974 | 0.7981613 | Chr1.4489, Chr1.4490, Chr1.4491 |
| Chr1 | 67150000 | 67160000 | -4.760941 | 0.7667219 | Chr1.4494, Chr1.4495, Chr1.4496 |
| Chr1 | 67155000 | 67165000 | -5.651528 | 0.7849797 | Chr1.4496, Chr1.4497, Chr1.4498 |
| Chr1 | 67170000 | 67180000 | -8.473701 | 0.8011622 | Chr1.4499, Chr1.4500 |
| Chr1 | 67220000 | 67230000 | -7.160649 | 0.8317691 | Chr1.4510, Chr1.4511 |
| Chr1 | 67225000 | 67235000 | -5.810928 | 0.8049447 | Chr1.4510, Chr1.4511, Chr1.4512, Chr1.4513 |
| Chr1 | 67240000 | 67250000 | -8.189343 | 0.7624402 | Chr1.4516, Chr1.4517, Chr1.4518, Chr1.4519 |
| Chr1 | 67245000 | 67255000 | -6.424644 | 0.846894 | Chr1.4517, Chr1.4518, Chr1.4519 |
| Chr1 | 67370000 | 67380000 | -8.060483 | 0.9454716 | Chr1.4547, Chr1.4548 |
| Chr1 | 67375000 | 67385000 | -8.864061 | 0.9456287 | Chr1.4548, Chr1.4549, Chr1.4550 |
| Chr2 | 11920000 | 11930000 | -10.08825 | 0.7698338 | Chr2.2322, Chr2.2323, Chr2.2324, Chr2.2325, Chr2.2326 |
| Chr2 | 11925000 | 11935000 | -5.594165 | 0.7736764 | Chr2.2325, Chr2.2326 |
| Chr2 | 11950000 | 11960000 | -6.235168 | 0.7968054 | Chr2.2328, Chr2.2329, Chr2.2330, Chr2.2331, Chr2.2332 |
| Chr2 | 11955000 | 11965000 | -10.41339 | 0.8638006 | Chr2.2330, Chr2.2331, Chr2.2332, Chr2.2333 |
| Chr2 | 11985000 | 11995000 | -5.627568 | 0.8802174 | Chr2.2337, Chr2.2338 |
| Chr2 | 11990000 | 12000000 | -10.55749 | 0.8542826 | Chr2.2337, Chr2.2338, Chr2.2339 |
| Chr2 | 11995000 | 12005000 | -8.680648 | 0.8556134 | Chr2.2339, Chr2.2340 |
| Chr2 | 12000000 | 12010000 | -8.419228 | 0.8534987 | Chr2.2340, Chr2.2341 |
| Chr2 | 12015000 | 12025000 | -6.637267 | 0.8598212 | Chr2.2343, Chr2.2344, Chr2.2345 |
| Chr2 | 12020000 | 12030000 | -5.07862 | 0.8402381 | Chr2.2344, Chr2.2345 |
| Chr2 | 12030000 | 12040000 | -4.453719 | 0.8431874 |  |
| Chr2 | 12055000 | 12065000 | -5.502643 | 0.8634098 | Chr2.2347, Chr2.2348, Chr2.2349, Chr2.2350, Chr2.2351, Chr2.2352 |
| Chr2 | 12130000 | 12140000 | -11.20588 | 0.8660476 | Chr2.2359, Chr2.2360, Chr2.2361 |
| Chr2 | 12135000 | 12145000 | -5.922589 | 0.8637607 | Chr2.2360, Chr2.2361, Chr2.2362, Chr2.2363 |
| Chr3 | 4400000 | 4410000 | -6.945989 | 0.756726 | Chr3.743, Chr3.744 |
| Chr3 | 51240000 | 51250000 | -4.786859 | 0.8242812 | Chr3.3952 |
| Chr3 | 51245000 | 51255000 | -6.356429 | 0.7871211 | Chr3.3953 |
| Chr3 | 51250000 | 51260000 | -6.524789 | 0.8483282 | Chr3.3953, Chr3.3954 |
| Chr3 | 51255000 | 51265000 | -8.86468 | 0.809575 | Chr3.3954, Chr3.3955 |
| Chr3 | 51260000 | 51270000 | -11.59437 | 0.831628 | Chr3.3954, Chr3.3955, Chr3.3956, Chr3.3957 |
| Chr3 | 51265000 | 51275000 | -6.603869 | 0.8403427 | Chr3.3956, Chr3.3957, Chr3.3958 |
| Chr3 | 51270000 | 51280000 | -6.976595 | 0.8408033 | Chr3.3957, Chr3.3958, Chr3.3959, Chr3.3960 |
| Chr3 | 51275000 | 51285000 | -15.02477 | 0.8443143 | Chr3.3959, Chr3.3960, Chr3.3961 |
| Chr3 | 51300000 | 51310000 | -12.56667 | 0.8698802 | Chr3.3966, Chr3.3967 |
| Chr3 | 51345000 | 51355000 | -9.16885 | 0.8194268 | Chr3.3972, Chr3.3973 |
| Chr3 | 51350000 | 51360000 | -8.712232 | 0.831914 | Chr3.3973, Chr3.3974 |
| Chr3 | 51355000 | 51365000 | -7.657706 | 0.8968783 | Chr3.3973, Chr3.3974, Chr3.3975 |
| Chr3 | 51395000 | 51405000 | -8.751513 | 0.7664009 | Chr3.3982, Chr3.3983 |
| Chr3 | 51425000 | 51435000 | -11.30207 | 0.8306556 | Chr3.3986 |
| Chr3 | 51430000 | 51440000 | -10.27014 | 0.8225919 | Chr3.3986, Chr3.3988 |
| Chr3 | 51435000 | 51445000 | -9.501823 | 0.8206074 | Chr3.3988, Chr3.3989, Chr3.3990 |
| Chr3 | 51440000 | 51450000 | -10.50067 | 0.7976779 | Chr3.3988, Chr3.3989, Chr3.3990, Chr3.3991 |
| Chr3 | 51445000 | 51455000 | -7.901578 | 0.8016179 | Chr3.3989, Chr3.3990, Chr3.3991 |
| Chr3 | 51450000 | 51460000 | -8.037069 | 0.8362076 | Chr3.3991, Chr3.3992 |
| Chr3 | 51455000 | 51465000 | -5.25245 | 0.847455 | Chr3.3992, Chr3.3993, Chr3.3994 |
| Chr3 | 51460000 | 51470000 | -5.032344 | 0.8717241 | Chr3.3993, Chr3.3994, Chr3.3995, Chr3.3996 |
| Chr3 | 51475000 | 51485000 | -11.87394 | 0.8884759 | Chr3.3998, Chr3.3999, Chr3.4000 |
| Chr3 | 51480000 | 51490000 | -9.956327 | 0.8699423 | Chr3.3999, Chr3.4000, Chr3.4001, Chr3.4002 |
| Chr3 | 51500000 | 51510000 | -8.254558 | 0.8608782 | Chr3.4007, Chr3.4008 |
| Chr3 | 51505000 | 51515000 | -8.697662 | 0.8818659 | Chr3.4008, Chr3.4009 |
| Chr3 | 51530000 | 51540000 | -4.405248 | 0.9153013 | Chr3.4014, Chr3.4015, Chr3.4016, Chr3.4017 |
| Chr3 | 51580000 | 51590000 | -5.022088 | 0.9315206 | Chr3.4023, Chr3.4024, Chr3.4025 |
| Chr3 | 51595000 | 51605000 | -7.905226 | 0.9121249 | Chr3.4027, Chr3.4028 |
| Chr3 | 51600000 | 51610000 | -7.351006 | 0.9505802 | Chr3.4028, Chr3.4029, Chr3.4030 |
| Chr3 | 51605000 | 51615000 | -9.554952 | 0.9500709 | Chr3.4029, Chr3.4030, Chr3.4031 |
| Chr3 | 51625000 | 51635000 | -9.241642 | 0.8623334 | Chr3.4032, Chr3.4033 |
| Chr3 | 51630000 | 51640000 | -6.794526 | 0.9470219 | Chr3.4032, Chr3.4033, Chr3.4034 |
| Chr3 | 51660000 | 51670000 | -9.283968 | 0.9404973 | Chr3.4039, Chr3.4040, Chr3.4041 |
| Chr3 | 51725000 | 51735000 | -9.821977 | 0.9314033 | Chr3.4057, Chr3.4058 |
| Chr3 | 51745000 | 51755000 | -7.840619 | 0.9504477 | Chr3.4064, Chr3.4065 |
| Chr3 | 53185000 | 53195000 | -10.03831 | 0.7700203 |  |
| Chr3 | 53205000 | 53215000 | -12.48529 | 0.8226816 | Chr3.4416, Chr3.4417, Chr3.4418 |
| Chr3 | 53210000 | 53220000 | -13.44316 | 0.8277254 | Chr3.4416, Chr3.4417, Chr3.4418, Chr3.4419, Chr3.4420, Chr3.4421 |
| Chr3 | 53215000 | 53225000 | -8.119591 | 0.8232374 | Chr3.4418, Chr3.4419, Chr3.4420, Chr3.4421, Chr3.4422 |
| Chr3 | 53220000 | 53230000 | -7.031542 | 0.8183076 | Chr3.4421, Chr3.4422, Chr3.4423 |
| Chr3 | 53225000 | 53235000 | -5.982052 | 0.8176408 | Chr3.4423 |
| Chr3 | 53230000 | 53240000 | -6.20456 | 0.8177355 | Chr3.4424 |
| Chr3 | 53235000 | 53245000 | -7.4036 | 0.8030201 | Chr3.4424 |
| Chr3 | 53240000 | 53250000 | -6.387941 | 0.7692832 | Chr3.4425 |
| Chr3 | 53270000 | 53280000 | -5.085546 | 0.7932802 | Chr3.4433, Chr3.4434, Chr3.4435 |
| Chr3 | 53275000 | 53285000 | -4.712601 | 0.8162108 | Chr3.4434, Chr3.4435, Chr3.4436, Chr3.4437 |
| Chr3 | 53280000 | 53290000 | -6.716942 | 0.8032748 | Chr3.4436, Chr3.4437, Chr3.4438, Chr3.4439 |
| Chr3 | 53285000 | 53295000 | -13.69664 | 0.8155622 | Chr3.4437, Chr3.4438, Chr3.4439, Chr3.4440 |
| Chr3 | 53290000 | 53300000 | -13.27211 | 0.8152723 | Chr3.4439, Chr3.4440, Chr3.4441 |
| Chr3 | 53295000 | 53305000 | -13.05616 | 0.8173289 | Chr3.4440, Chr3.4441, Chr3.4442, Chr3.4443 |
| Chr3 | 53300000 | 53310000 | -9.812661 | 0.8176074 | Chr3.4442, Chr3.4443, Chr3.4444 |
| Chr3 | 53320000 | 53330000 | -5.04161 | 0.842197 | Chr3.4448, Chr3.4449 |
| Chr3 | 53325000 | 53335000 | -6.607171 | 0.815852 | Chr3.4449, Chr3.4450 |
| Chr3 | 53345000 | 53355000 | -9.086054 | 0.8211465 | Chr3.4452, Chr3.4453, Chr3.4454 |
| Chr3 | 53350000 | 53360000 | -6.970482 | 0.8306722 | Chr3.4454 |
| Chr3 | 53355000 | 53365000 | -6.028994 | 0.8297942 |  |
| Chr3 | 53370000 | 53380000 | -7.539154 | 0.8249966 |  |
| Chr3 | 53395000 | 53405000 | -8.604598 | 0.8362741 | Chr3.4457_Chr3.4458 |
| Chr3 | 53400000 | 53410000 | -11.71138 | 0.8087039 | Chr3.4457_Chr3.4458, Chr3.4459 |
| Chr3 | 53405000 | 53415000 | -12.80375 | 0.855465 | Chr3.4459, Chr3.4460, Chr3.4461 |
| Chr3 | 53410000 | 53420000 | -10.04599 | 0.8398431 | Chr3.4460, Chr3.4461, Chr3.4462 |
| Chr4 | 2960000 | 2970000 | -11.01037 | 0.7512073 | Chr4.330, Chr4.331, Chr4.332, Chr4.333 |
| Chr4 | 4935000 | 4945000 | -12.51233 | 0.775717 | Chr4.831, Chr4.832, Chr4.833, Chr4.834 |
| Chr4 | 5005000 | 5015000 | -4.416936 | 0.7992743 | Chr4.848, Chr4.849, Chr4.850 |
| Chr4 | 5010000 | 5020000 | -5.566046 | 0.7994708 | Chr4.849, Chr4.850, Chr4.851, Chr4.852 |
| Chr4 | 5015000 | 5025000 | -5.104378 | 0.7969692 | Chr4.850, Chr4.851, Chr4.852 |
| Chr4 | 5020000 | 5030000 | -4.819515 | 0.7950327 | Chr4.852, Chr4.853, Chr4.854 |
| Chr4 | 5025000 | 5035000 | -5.936912 | 0.7976924 | Chr4.853, Chr4.854, Chr4.855 |
| Chr4 | 5030000 | 5040000 | -13.21524 | 0.8011595 | Chr4.854, Chr4.855, Chr4.856 |
| Chr4 | 7150000 | 7160000 | -6.932381 | 0.904284 | Chr4.1324, Chr4.1325, Chr4.1326 |
| Chr4 | 7610000 | 7620000 | -8.780996 | 0.9067353 | Chr4.1415, Chr4.1416, Chr4.1417, Chr4.1418, Chr4.1419 |
| Chr4 | 66330000 | 66340000 | -5.036567 | 0.7724304 | Chr4.4082, Chr4.4083, Chr4.4084 |
| Chr4 | 66355000 | 66365000 | -6.203399 | 0.8008271 | Chr4.4086 |
| Chr4 | 66410000 | 66420000 | -5.521995 | 0.8075098 |  |
| Chr4 | 66415000 | 66425000 | -5.95694 | 0.7961786 | Chr4.4092 |
| Chr4 | 66425000 | 66435000 | -4.406299 | 0.7954811 | Chr4.4092 |
| Chr4 | 66440000 | 66450000 | -4.837819 | 0.7669262 | Chr4.4094 |
| Chr4 | 66565000 | 66575000 | -5.010886 | 0.8069511 |  |
| Chr4 | 66630000 | 66640000 | -5.301024 | 0.7623296 | Chr4.4102, Chr4.4103 |
| Chr4 | 66635000 | 66645000 | -9.938742 | 0.8081299 | Chr4.4102, Chr4.4103, Chr4.4104, Chr4.4105, Chr4.4106 |
| Chr4 | 66640000 | 66650000 | -11.51795 | 0.8039978 | Chr4.4103, Chr4.4104, Chr4.4105, Chr4.4106 |
| Chr4 | 66645000 | 66655000 | -4.768571 | 0.7937676 | Chr4.4106, Chr4.4107, Chr4.4108 |
| Chr4 | 66935000 | 66945000 | -6.390902 | 0.7854349 | Chr4.4127 |
| Chr4 | 66960000 | 66970000 | -5.997112 | 0.8024103 |  |
| Chr4 | 66965000 | 66975000 | -5.169709 | 0.7917385 | Chr4.4131, Chr4.4132 |
| Chr5 | 4585000 | 4595000 | -7.270926 | 0.8782041 | Chr5.920 |
| Chr5 | 69435000 | 69445000 | -5.833945 | 0.7909709 | Chr5.5134, Chr5.5135, Chr5.5136 |
| Chr5 | 69465000 | 69475000 | -7.918037 | 0.8592589 | Chr5.5141, Chr5.5142 |
| Chr5 | 69470000 | 69480000 | -8.732764 | 0.9062182 | Chr5.5141, Chr5.5142, Chr5.5143 |
| Chr5 | 69505000 | 69515000 | -9.475233 | 0.7945068 | Chr5.5150, Chr5.5151, Chr5.5152, Chr5.5153 |
| Chr5 | 70015000 | 70025000 | -7.132389 | 0.8217424 | Chr5.5249, Chr5.5250, Chr5.5251 |
| Chr5 | 70155000 | 70165000 | -9.560559 | 0.852008 | Chr5.5285, Chr5.5286, Chr5.5287 |
| Chr5 | 70160000 | 70170000 | -9.31185 | 0.8620482 | Chr5.5286, Chr5.5287, Chr5.5288, Chr5.5289 |
| Chr5 | 70175000 | 70185000 | -10.8438 | 0.8108256 | Chr5.5290, Chr5.5291, Chr5.5292, Chr5.5293, Chr5.5294 |
| Chr5 | 70180000 | 70190000 | -12.00447 | 0.7950281 | Chr5.5293, Chr5.5294, Chr5.5295 |
| Chr5 | 70210000 | 70220000 | -4.608712 | 0.7720343 | Chr5.5300, Chr5.5301, Chr5.5302 |
| Chr5 | 70290000 | 70300000 | -9.561059 | 0.7554741 | Chr5.5317, Chr5.5318 |
| Chr5 | 70310000 | 70320000 | -6.195415 | 0.7853676 | Chr5.5321 |
| Chr5 | 70315000 | 70325000 | -5.506813 | 0.7932187 | Chr5.5321, Chr5.5322, Chr5.5323_Chr5.5324 |
| Chr6 | 55115000 | 55125000 | -6.681436 | 0.9060544 | Chr6.4258, Chr6.4259, Chr6.4260, Chr6.4261 |
| Chr6 | 55120000 | 55130000 | -8.044296 | 0.9055718 | Chr6.4259, Chr6.4260, Chr6.4261, Chr6.4262 |
| Chr6 | 55415000 | 55425000 | -12.88696 | 0.814494 | Chr6.4332 |
| Chr6 | 55430000 | 55440000 | -7.179901 | 0.899585 | Chr6.4333, Chr6.4334, Chr6.4335, Chr6.4336, Chr6.4337 |
| Chr6 | 55435000 | 55445000 | -9.413418 | 0.9030215 | Chr6.4336, Chr6.4337, Chr6.4338 |
| Chr6 | 55440000 | 55450000 | -8.649539 | 0.9020545 | Chr6.4337, Chr6.4338 |
| Chr6 | 55445000 | 55455000 | -7.636309 | 0.9032671 | Chr6.4340, Chr6.4341 |
| Chr6 | 55450000 | 55460000 | -4.578891 | 0.9241183 | Chr6.4340, Chr6.4341, Chr6.4342, Chr6.4343 |
| Chr6 | 55465000 | 55475000 | -4.791758 | 0.8965286 | Chr6.4344, Chr6.4345 |
| Chr6 | 55470000 | 55480000 | -4.788543 | 0.8975796 | Chr6.4345, Chr6.4346, Chr6.4347 |
| Chr6 | 55475000 | 55485000 | -4.515579 | 0.8980938 | Chr6.4346, Chr6.4347, Chr6.4348 |
| Chr6 | 55500000 | 55510000 | -8.719008 | 0.907566 | Chr6.4352, Chr6.4353 |
| Chr6 | 55520000 | 55530000 | -5.746716 | 0.8224824 | Chr6.4354, Chr6.4355, Chr6.4356 |
| Chr6 | 55550000 | 55560000 | -9.153515 | 0.9104499 | Chr6.4363, Chr6.4364_Chr6.4365, Chr6.4366, Chr6.4367 |
| Chr6 | 55555000 | 55565000 | -6.037444 | 0.9072216 | Chr6.4366, Chr6.4367, Chr6.4368, Chr6.4369 |
| Chr6 | 55560000 | 55570000 | -5.664096 | 0.9039956 | Chr6.4367, Chr6.4368, Chr6.4369, Chr6.4370, Chr6.4371 |
| Chr6 | 55575000 | 55585000 | -9.658989 | 0.9077917 | Chr6.4372, Chr6.4373, Chr6.4374 |
| Chr6 | 55580000 | 55590000 | -12.98361 | 0.9024799 | Chr6.4373, Chr6.4374, Chr6.4375, Chr6.4376 |
| Chr6 | 55585000 | 55595000 | -12.95178 | 0.9038086 | Chr6.4374, Chr6.4375, Chr6.4376, Chr6.4377, Chr6.4378 |
| Chr6 | 55590000 | 55600000 | -5.624899 | 0.9006345 | Chr6.4377, Chr6.4378 |
| Chr6 | 55595000 | 55605000 | -5.92452 | 0.9002728 | Chr6.4379, Chr6.4381 |
| Chr6 | 55600000 | 55610000 | -11.94273 | 0.9030887 | Chr6.4379, Chr6.4381, Chr6.4382, Chr6.4383 |
| Chr6 | 55605000 | 55615000 | -10.38726 | 0.9009728 | Chr6.4382, Chr6.4383, Chr6.4384 |
| Chr6 | 55610000 | 55620000 | -10.43141 | 0.8930415 | Chr6.4384 |
| Chr6 | 55615000 | 55625000 | -10.22791 | 0.8456958 | Chr6.4384, Chr6.4385, Chr6.4386 |
| Chr6 | 55825000 | 55835000 | -4.610833 | 0.8066346 | Chr6.4438, Chr6.4439 |
| Chr6 | 55830000 | 55840000 | -7.926353 | 0.8111944 | Chr6.4439, Chr6.4440 |
| Chr6 | 55835000 | 55845000 | -15.46686 | 0.8186782 | Chr6.4440, Chr6.4441 |
| Chr6 | 55840000 | 55850000 | -12.78849 | 0.8200288 | Chr6.4440, Chr6.4441, Chr6.4442, Chr6.4443 |
| Chr6 | 55845000 | 55855000 | -10.56635 | 0.8154365 | Chr6.4441, Chr6.4442, Chr6.4443, Chr6.4444 |
| Chr6 | 55860000 | 55870000 | -6.162907 | 0.7706313 | Chr6.4445, Chr6.4446, Chr6.4447 |
| Chr6 | 55865000 | 55875000 | -6.144343 | 0.773726 | Chr6.4447, Chr6.4448, Chr6.4449 |
| Chr6 | 55875000 | 55885000 | -11.29351 | 0.760868 | Chr6.4450, Chr6.4451, Chr6.4452, Chr6.4454 |
| Chr6 | 55880000 | 55890000 | -10.69731 | 0.8209596 | Chr6.4454, Chr6.4455 |
| Chr6 | 57240000 | 57250000 | -5.132216 | 0.7519199 | Chr6.4839, Chr6.4840, Chr6.4841, Chr6.4843, Chr6.4844 |
| Chr7 | 62650000 | 62660000 | -4.596657 | 0.9034742 | Chr7.3117, Chr7.3118, Chr7.3119, Chr7.3120 |
| Chr7 | 62655000 | 62665000 | -12.42379 | 0.888886 | Chr7.3118, Chr7.3119, Chr7.3120, Chr7.3121, Chr7.3122 |
| Chr7 | 62660000 | 62670000 | -12.14701 | 0.8813783 | Chr7.3120, Chr7.3121, Chr7.3122, Chr7.3123 |
| Chr7 | 62700000 | 62710000 | -8.332088 | 0.9067073 | Chr7.3128, Chr7.3129, Chr7.3130, Chr7.3131 |
| Chr7 | 62705000 | 62715000 | -7.760225 | 0.9102389 | Chr7.3129, Chr7.3130, Chr7.3131, Chr7.3132 |
| Chr7 | 62710000 | 62720000 | -6.814912 | 0.902328 | Chr7.3130, Chr7.3131, Chr7.3132, Chr7.3133, Chr7.3134 |
| Chr7 | 62715000 | 62725000 | -6.948586 | 0.901192 | Chr7.3132, Chr7.3133, Chr7.3134, Chr7.3135, Chr7.3136 |
| Chr7 | 62775000 | 62785000 | -9.409598 | 0.9284031 | Chr7.3144, Chr7.3145 |
| Chr7 | 62790000 | 62800000 | -4.619198 | 0.9286898 | Chr7.3146, Chr7.3147, Chr7.3148 |
| Chr7 | 62805000 | 62815000 | -7.567841 | 0.8751032 | Chr7.3149, Chr7.3150 |
| Chr7 | 62845000 | 62855000 | -9.153243 | 0.8937695 | Chr7.3155 |
| Chr7 | 62880000 | 62890000 | -9.313369 | 0.9395179 |  |
| Chr7 | 62885000 | 62895000 | -8.893612 | 0.9391034 |  |
| Chr7 | 62945000 | 62955000 | -7.697513 | 0.9353322 |  |
| Chr7 | 62960000 | 62970000 | -9.556599 | 0.9439609 | Chr7.3158, Chr7.3159 |
| Chr7 | 62965000 | 62975000 | -10.07982 | 0.9403662 | Chr7.3159, Chr7.3160 |
| Chr7 | 62990000 | 63000000 | -7.928235 | 0.9113895 | Chr7.3162 |
| Chr7 | 63040000 | 63050000 | -7.924504 | 0.8730577 | Chr7.3173 |
| Chr7 | 63045000 | 63055000 | -10.44282 | 0.9509347 |  |
| Chr7 | 63050000 | 63060000 | -10.97911 | 0.9493364 | Chr7.3174 |
| Chr7 | 63055000 | 63065000 | -10.89587 | 0.946574 | Chr7.3174, Chr7.3175 |
| Chr7 | 63070000 | 63080000 | -8.362819 | 0.9509574 | Chr7.3177, Chr7.3178, Chr7.3179, Chr7.3180, Chr7.3181, Chr7.3182 |
| Chr7 | 63100000 | 63110000 | -9.384136 | 0.9129155 | Chr7.3185, Chr7.3186, Chr7.3187 |
| Chr7 | 66860000 | 66870000 | -10.13624 | 0.7770528 | Chr7.4022, Chr7.4023, Chr7.4024 |
| Chr7 | 66875000 | 66885000 | -10.91804 | 0.8004844 | Chr7.4028, Chr7.4029, Chr7.4030, Chr7.4031, Chr7.4032, Chr7.4033 |
| Chr7 | 66880000 | 66890000 | -12.54399 | 0.7951858 | Chr7.4031, Chr7.4032, Chr7.4033, Chr7.4034, Chr7.4035 |
| Chr7 | 68120000 | 68130000 | -4.43692 | 0.8170178 | Chr7.4329, Chr7.4330, Chr7.4331 |
| Chr7 | 68135000 | 68145000 | -10.96095 | 0.8209325 | Chr7.4332, Chr7.4333, Chr7.4334, Chr7.4335 |
| Chr7 | 68140000 | 68150000 | -12.77994 | 0.8210985 | Chr7.4334, Chr7.4335, Chr7.4336 |
