# Supplementary material for: Genomic analysis of field pennycress (Thlaspi arvense) provides insights into mechanisms of adaptation to high elevation
Source: BMC Biol. 2021 Jul 22;19:143. doi: 10.1186/s12915-021-01079-0 (PMC8296595; doi:10.1186/s12915-021-01079-0)
Supplement: Supplementary file 15 — Additional file 15: Table S12. GO and KEGG functional categories of 359 candidate positively selected genes in HG based on top 3% cutoff of both FST(rms) and θπ ratio. [file 12915_2021_1079_MOESM15_ESM.docx]

**Table S12. GO and KEGG functional categories of 359 candidate positively selected genes in HG based on top 3% cutoff of both F_ST_(rms) and θπ ratio.**

| **GO category** | **P value** | **Num.** | **term** | **Genes** |
| --- | --- | --- | --- | --- |
| GO:0019367 | 2.85E-05 | 3 | fatty acid elongation | Chr6.4334, Chr6.4335, Chr6.4336 |
| GO:0048765 | 0.000161 | 8 | root hair cell differentiation | Chr2.2333, Chr2.2343, Chr4.4086, Chr5.5135, Chr5.5136, Chr5.5321, Chr6.4338, Chr6.4354 |
| GO:0071695 | 0.000189 | 10 | anatomical structure maturation | Chr2.2333, Chr2.2343, Chr4.4086, Chr5.5135, Chr5.5136, Chr5.5321, Chr6.4338, Chr6.4354, Chr6.4385, Chr6.4386 |
| GO:0016034 | 0.000205 | 2 | maleylacetoacetate isomerase activity | Chr7.3178, Chr7.3179 |
| GO:1905392 | 0.000217 | 16 | plant organ morphogenesis | Chr2.2330, Chr2.2333, Chr2.2343, Chr3.3975, Chr3.4425, Chr4.4086, Chr5.5135, Chr5.5136, Chr5.5150, Chr5.5321, Chr6.4338, Chr6.4354, Chr6.4385, Chr6.4386, Chr7.3134, Chr7.3149 |
| GO:1901616 | 0.000349 | 4 | organic hydroxy compound catabolic process | Chr6.4385, Chr6.4386, Chr7.3178, Chr7.3179 |
| GO:0030176 | 0.000576 | 6 | integral component of endoplasmic reticulum membrane | Chr3.3994, Chr6.4258, Chr6.4334, Chr6.4335, Chr6.4336, Chr6.4352 |
| GO:1901068 | 0.000581 | 3 | guanosine-containing compound metabolic process | Chr3.4462, Chr5.5300, Chr6.4366 |
| GO:0044109 | 0.000608 | 2 | cellular alcohol catabolic process | Chr6.4385, Chr6.4386 |
| GO:0061412 | 0.000608 | 2 | positive regulation of transcription from RNA polymerase II promoter in response to amino acid starvation | Chr6.4385, Chr6.4386 |
| GO:0070786 | 0.000608 | 2 | positive regulation of growth of unicellular organism as a thread of attached cells | Chr6.4385, Chr6.4386 |
| GO:0090033 | 0.000608 | 2 | positive regulation of filamentous growth | Chr6.4385, Chr6.4386 |
| GO:1900189 | 0.000608 | 2 | positive regulation of cell adhesion involved in single-species biofilm formation | Chr6.4385, Chr6.4386 |
| GO:1900192 | 0.000608 | 2 | positive regulation of single-species biofilm formation | Chr6.4385, Chr6.4386 |
| GO:1900430 | 0.000608 | 2 | positive regulation of filamentous growth of a population of unicellular organisms | Chr6.4385, Chr6.4386 |
| GO:2000219 | 0.000608 | 2 | positive regulation of invasive growth in response to glucose limitation | Chr6.4385, Chr6.4386 |
| GO:0021700 | 0.000628 | 10 | developmental maturation | Chr2.2333, Chr2.2343, Chr4.4086, Chr5.5135, Chr5.5136, Chr5.5321, Chr6.4338, Chr6.4354, Chr6.4385, Chr6.4386 |
| GO:0015934 | 0.000631 | 8 | large ribosomal subunit | Chr1.4549, Chr2.2332, Chr3.4002, Chr4.4108, Chr6.4261, Chr6.4363, Chr6.4447, Chr6.4452 |
| GO:0031227 | 0.000716 | 6 | intrinsic component of endoplasmic reticulum membrane | Chr3.3994, Chr6.4258, Chr6.4334, Chr6.4335, Chr6.4336, Chr6.4352 |
| GO:0042766 | 0.001205 | 2 | nucleosome mobilization | Chr6.4385, Chr6.4386 |
| GO:1901999 | 0.001205 | 2 | homogentisate metabolic process | Chr7.3178, Chr7.3179 |
| GO:0031224 | 0.001206 | 25 | intrinsic component of membrane | Chr1.3983, Chr1.4516, Chr1.4517, Chr3.3967, Chr3.3994, Chr3.4433, Chr3.4434, Chr3.4435, Chr3.4436, Chr3.4442, Chr3.4443, Chr4.1419, Chr5.5134, Chr5.5295, Chr5.5302, Chr6.4258, Chr6.4334, Chr6.4335, Chr6.4336, Chr6.4337, Chr6.4352, Chr6.4375, Chr6.4381, Chr6.4844, Chr7.3119 |
| GO:0080147 | 0.001221 | 6 | root hair cell development | Chr4.4086, Chr5.5135, Chr5.5136, Chr5.5321, Chr6.4338, Chr6.4354 |
| GO:0090627 | 0.001386 | 8 | plant epidermal cell differentiation | Chr2.2333, Chr2.2343, Chr4.4086, Chr5.5135, Chr5.5136, Chr5.5321, Chr6.4338, Chr6.4354 |
| GO:0090558 | 0.001388 | 11 | plant epidermis development | Chr1.4498, Chr2.2333, Chr2.2343, Chr4.4086, Chr5.5135, Chr5.5136, Chr5.5321, Chr6.4338, Chr6.4354, Chr6.4373, Chr7.3185 |
| GO:0009922 | 0.001418 | 3 | fatty acid elongase activity | Chr6.4334, Chr6.4335, Chr6.4336 |
| GO:0010015 | 0.001431 | 11 | root morphogenesis | Chr2.2333, Chr2.2343, Chr4.4086, Chr5.5135, Chr5.5136, Chr5.5321, Chr6.4338, Chr6.4354, Chr6.4385, Chr6.4386, Chr7.3134 |
| GO:0022625 | 0.001553 | 7 | cytosolic large ribosomal subunit | Chr1.4549, Chr2.2332, Chr3.4002, Chr6.4261, Chr6.4363, Chr6.4447, Chr6.4452 |
| GO:0004385 | 0.001989 | 2 | guanylate kinase activity | Chr3.4462, Chr6.4366 |
| GO:0006076 | 0.001989 | 2 | (1->3)-beta-D-glucan catabolic process | Chr3.4434, Chr3.4436 |
| GO:0044107 | 0.001989 | 2 | cellular alcohol metabolic process | Chr6.4385, Chr6.4386 |
| GO:0046710 | 0.001989 | 2 | GDP metabolic process | Chr3.4462, Chr6.4366 |
| GO:0051275 | 0.001989 | 2 | beta-glucan catabolic process | Chr3.4434, Chr3.4436 |
| GO:0030497 | 0.002024 | 3 | fatty acid elongation | Chr6.4334, Chr6.4335, Chr6.4336 |
| GO:0040008 | 0.002253 | 10 | regulation of growth | Chr3.3975, Chr3.4450, Chr3.4462, Chr5.5302, Chr6.4366, Chr6.4384, Chr6.4385, Chr6.4386, Chr6.4440, Chr7.3134 |
| GO:0009888 | 0.002386 | 19 | tissue development | Chr1.4498, Chr2.2330, Chr2.2333, Chr2.2343, Chr3.3975, Chr3.4425, Chr4.4086, Chr5.5135, Chr5.5136, Chr5.5141, Chr5.5321, Chr6.4338, Chr6.4354, Chr6.4373, Chr6.4384, Chr6.4385, Chr6.4386, Chr7.3134, Chr7.3185 |
| GO:0010051 | 0.002716 | 5 | xylem and phloem pattern formation | Chr2.2330, Chr3.3975, Chr5.5321, Chr7.3132, Chr7.3150 |
| GO:0006074 | 0.002956 | 2 | (1->3)-beta-D-glucan metabolic process | Chr3.4434, Chr3.4436 |
| GO:0009921 | 0.002956 | 2 | auxin efflux carrier complex | Chr5.5135, Chr5.5136 |
| GO:0036003 | 0.002956 | 2 | positive regulation of transcription from RNA polymerase II promoter in response to stress | Chr6.4385, Chr6.4386 |
| GO:0046037 | 0.002956 | 2 | GMP metabolic process | Chr3.4462, Chr6.4366 |
| GO:0010073 | 0.003192 | 7 | meristem maintenance | Chr3.3975, Chr3.4425, Chr5.5141, Chr6.4384, Chr6.4385, Chr6.4386, Chr7.3134 |
| GO:0006261 | 0.003654 | 6 | DNA-dependent DNA replication | Chr3.4422, Chr6.4385, Chr6.4386, Chr6.4439, Chr7.3185, Chr7.4329 |
| GO:0007389 | 0.003776 | 9 | pattern specification process | Chr2.2330, Chr3.3975, Chr3.4425, Chr5.5135, Chr5.5136, Chr5.5150, Chr5.5321, Chr7.3132, Chr7.3150 |
| GO:0016021 | 0.003933 | 17 | integral component of membrane | Chr1.4516, Chr3.3967, Chr3.3994, Chr3.4433, Chr3.4442, Chr3.4443, Chr4.1419, Chr5.5134, Chr5.5295, Chr5.5302, Chr6.4258, Chr6.4334, Chr6.4335, Chr6.4336, Chr6.4337, Chr6.4352, Chr6.4375 |
| GO:0001102 | 0.004099 | 2 | RNA polymerase II activating transcription factor binding | Chr6.4385, Chr6.4386 |
| GO:0010455 | 0.004099 | 2 | positive regulation of cell fate commitment | Chr6.4385, Chr6.4386 |
| GO:0019336 | 0.004099 | 2 | phenol-containing compound catabolic process | Chr7.3178, Chr7.3179 |
| GO:0031492 | 0.004099 | 2 | nucleosomal DNA binding | Chr6.4385, Chr6.4386 |
| GO:0045785 | 0.004099 | 2 | positive regulation of cell adhesion | Chr6.4385, Chr6.4386 |
| GO:1901070 | 0.004099 | 2 | guanosine-containing compound biosynthetic process | Chr3.4462, Chr5.5300 |
| GO:0044445 | 0.004502 | 11 | cytosolic part | Chr1.3983, Chr1.4549, Chr2.2332, Chr3.4002, Chr3.4438, Chr6.4261, Chr6.4363, Chr6.4447, Chr6.4452, Chr6.4455, Chr7.3136 |
| GO:0044391 | 0.004795 | 9 | ribosomal subunit | Chr1.4549, Chr2.2332, Chr3.4002, Chr4.4108, Chr6.4261, Chr6.4363, Chr6.4447, Chr6.4452, Chr7.3136 |
| GO:0004004 | 0.004823 | 6 | ATP-dependent RNA helicase activity | Chr2.2362, Chr2.2363, Chr3.4014, Chr3.4015, Chr5.5321, Chr6.4333 |
| GO:0008186 | 0.004823 | 6 | RNA-dependent ATPase activity | Chr2.2362, Chr2.2363, Chr3.4014, Chr3.4015, Chr5.5321, Chr6.4333 |
| GO:0003724 | 0.005042 | 6 | RNA helicase activity | Chr2.2362, Chr2.2363, Chr3.4014, Chr3.4015, Chr5.5321, Chr6.4333 |
| GO:0046128 | 0.005298 | 3 | purine ribonucleoside metabolic process | Chr3.4462, Chr5.5300, Chr6.4366 |
| GO:0000182 | 0.005414 | 2 | rDNA binding | Chr6.4385, Chr6.4386 |
| GO:0010810 | 0.005414 | 2 | regulation of cell-substrate adhesion | Chr6.4385, Chr6.4386 |
| GO:0016328 | 0.005414 | 2 | lateral plasma membrane | Chr5.5135, Chr5.5136 |
| GO:0016514 | 0.005414 | 2 | SWI/SNF complex | Chr6.4385, Chr6.4386 |
| GO:0070577 | 0.005414 | 2 | lysine-acetylated histone binding | Chr6.4385, Chr6.4386 |
| GO:0140033 | 0.005414 | 2 | acetylation-dependent protein binding | Chr6.4385, Chr6.4386 |
| GO:0042278 | 0.005927 | 3 | purine nucleoside metabolic process | Chr3.4462, Chr5.5300, Chr6.4366 |
| GO:0042761 | 0.005927 | 3 | very long-chain fatty acid biosynthetic process | Chr6.4334, Chr6.4335, Chr6.4336 |
| GO:0046467 | 0.006136 | 5 | membrane lipid biosynthetic process | Chr6.4258, Chr6.4334, Chr6.4335, Chr6.4336, Chr6.4441 |
| GO:0009965 | 0.006796 | 5 | leaf morphogenesis | Chr2.2330, Chr2.2333, Chr3.3975, Chr3.4425, Chr7.3149 |
| GO:0010231 | 0.006895 | 2 | maintenance of seed dormancy | Chr6.4385, Chr6.4386 |
| GO:0031901 | 0.006895 | 2 | early endosome membrane | Chr2.2324, Chr7.3121 |
| GO:0048577 | 0.006895 | 2 | negative regulation of short-day photoperiodism, flowering | Chr1.4500, Chr3.4448 |
| GO:0097437 | 0.006895 | 2 | maintenance of dormancy | Chr6.4385, Chr6.4386 |
| GO:0042623 | 0.007245 | 13 | ATPase activity, coupled | Chr1.4550, Chr2.2362, Chr2.2363, Chr3.4014, Chr3.4015, Chr3.4442, Chr3.4443, Chr5.5317, Chr5.5321, Chr6.4333, Chr6.4385, Chr6.4386, Chr6.4455 |
| GO:0004312 | 0.007313 | 3 | fatty acid synthase activity | Chr6.4334, Chr6.4335, Chr6.4336 |
| GO:0009313 | 0.008072 | 3 | oligosaccharide catabolic process | Chr3.4457_Chr3.4458, Chr6.4385, Chr6.4386 |
| GO:0010305 | 0.008072 | 3 | leaf vascular tissue pattern formation | Chr2.2330, Chr3.3975, Chr7.3150 |
| GO:0031597 | 0.008538 | 2 | cytosolic proteasome complex | Chr3.4438, Chr6.4455 |
| GO:0034198 | 0.008538 | 2 | cellular response to amino acid starvation | Chr6.4385, Chr6.4386 |
| GO:0043618 | 0.008538 | 2 | regulation of transcription from RNA polymerase II promoter in response to stress | Chr6.4385, Chr6.4386 |
| GO:1990928 | 0.008538 | 2 | response to amino acid starvation | Chr6.4385, Chr6.4386 |
| GO:0000038 | 0.009724 | 3 | very long-chain fatty acid metabolic process | Chr6.4334, Chr6.4335, Chr6.4336 |
| GO:0048364 | 0.009963 | 14 | root development | Chr2.2333, Chr2.2343, Chr4.4086, Chr5.5135, Chr5.5136, Chr5.5321, Chr6.4337, Chr6.4338, Chr6.4354, Chr6.4385, Chr6.4386, Chr6.4441, Chr7.3134, Chr7.3162 |
| **KEGG category** | **P value** | **Num** | **term** | **Genes** |
| ko00604 | 0.000706 | 3 | Glycosphingolipid biosynthesis - ganglio series | Chr3.3982, Chr3.4007, Chr3.4009 |
| ko00531 | 0.001692 | 3 | Glycosaminoglycan degradation | Chr3.3982, Chr3.4007, Chr3.4009 |
| ko00511 | 0.004084 | 3 | Other glycan degradation | Chr3.3982, Chr3.4007, Chr3.4009 |
| ko01040 | 0.005523 | 3 | Biosynthesis of unsaturated fatty acids | Chr6.4334, Chr6.4335, Chr6.4336 |
| ko00062 | 0.014998 | 3 | Fatty acid elongation | Chr6.4334, Chr6.4335, Chr6.4336 |
| ko00052 | 0.02018 | 4 | Galactose metabolism | Chr3.3982, Chr3.4007, Chr3.4009, Chr3.4457_Chr3.4458 |
| ko00600 | 0.02126 | 3 | Sphingolipid metabolism | Chr3.3982, Chr3.4007, Chr3.4009 |
| ko00350 | 0.030118 | 3 | Tyrosine metabolism | Chr7.3177, Chr7.3178, Chr7.3179 |
