# Supplementary material for: Genomic analysis of field pennycress (Thlaspi arvense) provides insights into mechanisms of adaptation to high elevation
Source: BMC Biol. 2021 Jul 22;19:143. doi: 10.1186/s12915-021-01079-0 (PMC8296595; doi:10.1186/s12915-021-01079-0)
Supplement: Supplementary file 16 — Additional file 16: Table S13. GO and KEGG functional categories of 621 candidate positively selected genes in HG based on the CLR test. [file 12915_2021_1079_MOESM16_ESM.docx]

**Table S13. GO and KEGG functional categories of 621 candidate positively selected genes in HG based on CLR test.**

| **GO category** | **P value** | **Num** | **term** | **Genes** |
| --- | --- | --- | --- | --- |
| GO:0009187 | 0.000116 | 4 | cyclic nucleotide metabolic process | Chr2.2302, Chr2.2303, Chr2.2304, Chr7.3173 |
| GO:0046827 | 0.000523 | 2 | positive regulation of protein export from nucleus | Chr2.2333, Chr3.4003 |
| GO:1900378 | 0.000612 | 3 | positive regulation of secondary metabolite biosynthetic process | Chr3.4021, Chr3.4406, Chr3.4407 |
| GO:1900745 | 0.000612 | 3 | positive regulation of p38MAPK cascade | Chr4.871, Chr4.873, Chr4.880 |
| GO:0051973 | 0.000641 | 4 | positive regulation of telomerase activity | Chr3.4082, Chr4.871, Chr4.873, Chr4.880 |
| GO:0032153 | 0.000842 | 6 | cell division site | Chr3.4003, Chr3.4076, Chr4.871, Chr4.873, Chr4.880, Chr4.925 |
| GO:0043506 | 0.000903 | 3 | regulation of JUN kinase activity | Chr4.871, Chr4.873, Chr4.880 |
| GO:0046461 | 0.000903 | 3 | neutral lipid catabolic process | Chr2.2353, Chr3.4426, Chr3.4427 |
| GO:0046464 | 0.000903 | 3 | acylglycerol catabolic process | Chr2.2353, Chr3.4426, Chr3.4427 |
| GO:0032880 | 0.000982 | 8 | regulation of protein localization | Chr2.2293, Chr2.2333, Chr3.4003, Chr3.4078, Chr3.4079, Chr4.847, Chr4.915, Chr4.925 |
| GO:0046822 | 0.001188 | 4 | regulation of nucleocytoplasmic transport | Chr2.2293, Chr2.2333, Chr3.4003, Chr4.847 |
| GO:0032874 | 0.001268 | 3 | positive regulation of stress-activated MAPK cascade | Chr4.871, Chr4.873, Chr4.880 |
| GO:0010225 | 0.001426 | 4 | response to UV-C | Chr4.871, Chr4.873, Chr4.880, Chr7.3164 |
| GO:0032155 | 0.00146 | 5 | cell division site part | Chr3.4076, Chr4.871, Chr4.873, Chr4.880, Chr4.925 |
| GO:0004445 | 0.001545 | 2 | inositol-polyphosphate 5-phosphatase activity | Chr2.2429, Chr2.2430 |
| GO:1900744 | 0.001714 | 3 | regulation of p38MAPK cascade | Chr4.871, Chr4.873, Chr4.880 |
| GO:1904353 | 0.001714 | 3 | regulation of telomere capping | Chr4.871, Chr4.873, Chr4.880 |
| GO:0055072 | 0.002596 | 5 | iron ion homeostasis | Chr2.2626, Chr3.4406, Chr3.4407, Chr4.904, Chr7.3135 |
| GO:2000573 | 0.002713 | 4 | positive regulation of DNA biosynthetic process | Chr3.4082, Chr4.871, Chr4.873, Chr4.880 |
| GO:0000935 | 0.002871 | 3 | division septum | Chr4.871, Chr4.873, Chr4.880 |
| GO:0006388 | 0.002871 | 3 | tRNA splicing, via endonucleolytic cleavage and ligation | Chr2.2302, Chr2.2303, Chr2.2304 |
| GO:0030428 | 0.002871 | 3 | cell septum | Chr4.871, Chr4.873, Chr4.880 |
| GO:0042973 | 0.002871 | 3 | glucan endo-1,3-beta-D-glucosidase activity | Chr2.2387, Chr3.4434, Chr3.4436 |
| GO:0051223 | 0.002887 | 6 | regulation of protein transport | Chr2.2293, Chr2.2333, Chr3.4003, Chr3.4078, Chr3.4079, Chr4.847 |
| GO:0004351 | 0.003044 | 2 | glutamate decarboxylase activity | Chr7.3155, Chr7.3156 |
| GO:0019433 | 0.003044 | 2 | triglyceride catabolic process | Chr3.4426, Chr3.4427 |
| GO:0032957 | 0.003044 | 2 | inositol trisphosphate metabolic process | Chr2.2429, Chr2.2430 |
| GO:1900704 | 0.003044 | 2 | regulation of siderophore biosynthetic process | Chr3.4406, Chr3.4407 |
| GO:1900706 | 0.003044 | 2 | positive regulation of siderophore biosynthetic process | Chr3.4406, Chr3.4407 |
| GO:0051972 | 0.003128 | 4 | regulation of telomerase activity | Chr3.4082, Chr4.871, Chr4.873, Chr4.880 |
| GO:0001871 | 0.00313 | 6 | obsolete pattern binding | Chr2.2387, Chr3.4434, Chr3.4435, Chr3.4436, Chr4.841, Chr7.3119 |
| GO:0030247 | 0.00313 | 6 | polysaccharide binding | Chr2.2387, Chr3.4434, Chr3.4435, Chr3.4436, Chr4.841, Chr7.3119 |
| GO:0070201 | 0.00313 | 6 | regulation of establishment of protein localization | Chr2.2293, Chr2.2333, Chr3.4003, Chr3.4078, Chr3.4079, Chr4.847 |
| GO:0042578 | 0.003146 | 14 | phosphoric ester hydrolase activity | Chr2.2302, Chr2.2303, Chr2.2304, Chr2.2349, Chr2.2429, Chr2.2430, Chr3.4107, Chr4.919, Chr4.926, Chr7.3139, Chr7.3142, Chr7.3143, Chr7.3144, Chr7.3145 |
| GO:0005938 | 0.003193 | 9 | cell cortex | Chr2.2314, Chr3.4376, Chr4.833, Chr4.836, Chr4.871, Chr4.873, Chr4.880, Chr4.925, Chr7.3132 |
| GO:0006879 | 0.003584 | 4 | cellular iron ion homeostasis | Chr3.4406, Chr3.4407, Chr4.904, Chr7.3135 |
| GO:0032212 | 0.003593 | 3 | positive regulation of telomere maintenance via telomerase | Chr4.871, Chr4.873, Chr4.880 |
| GO:0046503 | 0.003593 | 3 | glycerolipid catabolic process | Chr2.2353, Chr3.4426, Chr3.4427 |
| GO:0051194 | 0.003593 | 3 | positive regulation of cofactor metabolic process | Chr3.4406, Chr3.4407, Chr3.4430 |
| GO:1904358 | 0.003593 | 3 | positive regulation of telomere maintenance via telomere lengthening | Chr4.871, Chr4.873, Chr4.880 |
| GO:0009755 | 0.004411 | 22 | hormone-mediated signaling pathway | Chr2.2333, Chr2.2345, Chr2.2366, Chr2.2376, Chr3.4002, Chr3.4104, Chr3.4376, Chr3.4387, Chr3.4395, Chr3.4414, Chr3.4425, Chr3.4430, Chr4.836, Chr4.853, Chr4.859, Chr4.900, Chr4.915, Chr4.919, Chr7.3112, Chr7.3134, Chr7.3139, Chr7.3150 |
| GO:0004439 | 0.004415 | 3 | phosphatidylinositol-4,5-bisphosphate 5-phosphatase activity | Chr2.2429, Chr2.2430, Chr7.3145 |
| GO:0046328 | 0.004415 | 3 | regulation of JNK cascade | Chr4.871, Chr4.873, Chr4.880 |
| GO:0106019 | 0.004415 | 3 | phosphatidylinositol-4,5-bisphosphate phosphatase activity | Chr2.2429, Chr2.2430, Chr7.3145 |
| GO:0006076 | 0.004996 | 2 | (1->3)-beta-D-glucan catabolic process | Chr3.4434, Chr3.4436 |
| GO:0046030 | 0.004996 | 2 | inositol trisphosphate phosphatase activity | Chr2.2429, Chr2.2430 |
| GO:0051275 | 0.004996 | 2 | beta-glucan catabolic process | Chr3.4434, Chr3.4436 |
| GO:0000394 | 0.005343 | 3 | RNA splicing, via endonucleolytic cleavage and ligation | Chr2.2302, Chr2.2303, Chr2.2304 |
| GO:0032872 | 0.005343 | 3 | regulation of stress-activated MAPK cascade | Chr4.871, Chr4.873, Chr4.880 |
| GO:0033135 | 0.005343 | 3 | regulation of peptidyl-serine phosphorylation | Chr2.2307, Chr2.2308, Chr4.847 |
| GO:0009627 | 0.005574 | 5 | systemic acquired resistance | Chr2.2299, Chr2.2365, Chr3.4435, Chr4.853, Chr4.858 |
| GO:0032386 | 0.005574 | 5 | regulation of intracellular transport | Chr2.2293, Chr2.2333, Chr3.4003, Chr4.847, Chr7.3097 |
| GO:0099568 | 0.005938 | 9 | cytoplasmic region | Chr2.2314, Chr3.4376, Chr4.833, Chr4.836, Chr4.871, Chr4.873, Chr4.880, Chr4.925, Chr7.3132 |
| GO:0043622 | 0.006066 | 5 | cortical microtubule organization | Chr3.4036, Chr3.4076, Chr4.871, Chr4.873, Chr4.880 |
| GO:0034594 | 0.006379 | 3 | phosphatidylinositol trisphosphate phosphatase activity | Chr2.2429, Chr2.2430, Chr7.3145 |
| GO:1905622 | 0.006379 | 3 | negative regulation of leaf development | Chr2.2288, Chr4.853, Chr7.3112 |
| GO:0042176 | 0.006486 | 6 | regulation of protein catabolic process | Chr3.4025, Chr3.4078, Chr3.4079, Chr3.4081, Chr3.4097, Chr7.3112 |
| GO:0006779 | 0.006549 | 4 | porphyrin-containing compound biosynthetic process | Chr2.2361, Chr3.4403, Chr4.881, Chr4.895 |
| GO:0006074 | 0.007381 | 2 | (1->3)-beta-D-glucan metabolic process | Chr3.4434, Chr3.4436 |
| GO:0034497 | 0.007381 | 2 | protein localization to phagophore assembly site | Chr3.4357, Chr4.845 |
| GO:0000186 | 0.007527 | 3 | activation of MAPKK activity | Chr4.871, Chr4.873, Chr4.880 |
| GO:0004709 | 0.007527 | 3 | MAP kinase kinase kinase activity | Chr4.871, Chr4.873, Chr4.880 |
| GO:0010453 | 0.007527 | 3 | regulation of cell fate commitment | Chr2.2404, Chr2.2405, Chr7.3112 |
| GO:0046685 | 0.007527 | 3 | response to arsenic-containing substance | Chr2.2306, Chr3.4406, Chr3.4407 |
| GO:0030246 | 0.007656 | 7 | carbohydrate binding | Chr2.2387, Chr3.4000, Chr3.4434, Chr3.4435, Chr3.4436, Chr4.841, Chr7.3119 |
| GO:0046486 | 0.007697 | 9 | glycerolipid metabolic process | Chr2.2353, Chr2.2429, Chr2.2430, Chr3.4405, Chr3.4426, Chr3.4427, Chr3.4429, Chr4.858, Chr7.3145 |
| GO:0033014 | 0.008086 | 4 | tetrapyrrole biosynthetic process | Chr2.2361, Chr3.4403, Chr4.881, Chr4.895 |
| GO:0033157 | 0.008086 | 4 | regulation of intracellular protein transport | Chr2.2293, Chr2.2333, Chr3.4003, Chr4.847 |
| GO:0015995 | 0.008788 | 3 | chlorophyll biosynthetic process | Chr2.2361, Chr3.4403, Chr4.895 |
| GO:0032206 | 0.008788 | 3 | positive regulation of telomere maintenance | Chr4.871, Chr4.873, Chr4.880 |
| GO:0044247 | 0.008936 | 4 | cellular polysaccharide catabolic process | Chr3.4434, Chr3.4436, Chr4.841, Chr4.915 |
| GO:1903827 | 0.008993 | 5 | regulation of cellular protein localization | Chr2.2293, Chr2.2333, Chr3.4003, Chr4.847, Chr4.925 |
| GO:0000272 | 0.009678 | 5 | polysaccharide catabolic process | Chr2.2403, Chr3.4434, Chr3.4436, Chr4.841, Chr4.915 |
| **KEGG category** | **P value** | **Num** | **term** | **Genes** |
| ko00604 | 5.77E-06 | 5 | Glycosphingolipid biosynthesis - ganglio series | Chr3.4005, Chr3.4006, Chr3.4007, Chr3.4009, Chr3.4010 |
| ko00531 | 2.76E-05 | 5 | Glycosaminoglycan degradation | Chr3.4005, Chr3.4006, Chr3.4007, Chr3.4009, Chr3.4010 |
| ko00511 | 0.000129 | 5 | Other glycan degradation | Chr3.4005, Chr3.4006, Chr3.4007, Chr3.4009, Chr3.4010 |
| ko00600 | 0.000292 | 6 | Sphingolipid metabolism | Chr3.4005, Chr3.4006, Chr3.4007, Chr3.4009, Chr3.4010, Chr3.4103 |
| ko00740 | 0.005125 | 3 | Riboflavin metabolism | Chr7.3142, Chr7.3143, Chr7.3144 |
| ko04075 | 0.006627 | 14 | Plant hormone signal transduction | Chr2.2333, Chr2.2345, Chr2.2431, Chr2.2432, Chr3.4029, Chr3.4104, Chr3.4393, Chr3.4414, Chr3.4425, Chr4.859, Chr4.900, Chr7.3112, Chr7.3134, Chr7.3139 |
| ko00250 | 0.015357 | 4 | Alanine, aspartate and glutamate metabolism | Chr2.2367, Chr3.4021, Chr7.3155, Chr7.3156 |
| ko00052 | 0.023019 | 5 | Galactose metabolism | Chr3.4005, Chr3.4006, Chr3.4007, Chr3.4009, Chr3.4010 |
| ko00510 | 0.023579 | 4 | N-Glycan biosynthesis | Chr2.2398, Chr3.4433, Chr4.917, Chr7.3128 |
| ko00430 | 0.031697 | 2 | Taurine and hypotaurine metabolism | Chr7.3155, Chr7.3156 |
| ko04141 | 0.03424 | 10 | Protein processing in endoplasmic reticulum | Chr3.4025, Chr3.4081, Chr3.4105, Chr3.4115, Chr3.4423, Chr3.4444, Chr4.905, Chr7.3097, Chr7.3121, Chr7.3128 |
| ko01502 | 0.037445 | 1 | Vancomycin resistance | Chr2.2391 |
