# Supplementary material for: Genomic analysis of field pennycress (Thlaspi arvense) provides insights into mechanisms of adaptation to high elevation
Source: BMC Biol. 2021 Jul 22;19:143. doi: 10.1186/s12915-021-01079-0 (PMC8296595; doi:10.1186/s12915-021-01079-0)
Supplement: Supplementary file 17 — Additional file 17: Table S14. List of 130 positively selected genes in HG based on three selection tests [FST(rms), θπ ratio and CLR]. [file 12915_2021_1079_MOESM17_ESM.docx]

**Table S14. List of 130 positively selected genes in HG based on three selection tests [F_ST_(rms), θπ ratio and CLR].**

| 130 PSGs | Annotation in NR database |
| --- | --- |
| Chr1.2322 | uncharacterized protein LOC18030499 isoform X2 [Eutrema salsugineum] |
| Chr1.2323 | BnaCnng70620D [Brassica napus] |
| Chr1.2324 | RAB GTPase homolog A1D [Arabidopsis thaliana] |
| Chr1.2325 | metal transporter Nramp5 [Arabidopsis lyrata subsp. lyrata] |
| Chr1.2326 | cellulose synthase A catalytic subunit 8 [UDP-forming] [Eutrema salsugineum] |
| Chr1.2328 | transcription factor MYB98 [Eutrema salsugineum] |
| Chr1.2329 | receptor-like protein 51 [Eutrema salsugineum] |
| Chr1.2330 | pentatricopeptide repeat-containing protein DOT4, chloroplastic [Eutrema salsugineum] |
| Chr1.2331 | SAP-like protein BP-73 isoform X1 [Eutrema salsugineum] |
| Chr1.2332 | PREDICTED: 60S ribosomal protein L11-1-like [Brassica rapa] |
| Chr1.2333 | unnamed protein product [Brassica rapa] |
| Chr1.2337 | hypothetical protein EUTSA_v10024510mg [Eutrema salsugineum] |
| Chr1.2338 | hypothetical protein [Arabidopsis thaliana] |
| Chr1.2339 | protein DOG1-like 1 [Eutrema salsugineum] |
| Chr1.2340 | PREDICTED: uncharacterized protein LOC108840972 [Raphanus sativus] |
| Chr1.2341 | PREDICTED: transcription factor TGAL3 [Raphanus sativus] |
| Chr1.2343 | protein MALE DISCOVERER 2 [Eutrema salsugineum] |
| Chr1.2344 | uncharacterized protein LOC18030518 [Eutrema salsugineum] |
| Chr1.2345 | PREDICTED: abscisic acid receptor PYL13 [Brassica oleracea var. oleracea] |
| Chr1.2347 | scar-like domain-containing protein WAVE 5 isoform X1 [Eutrema salsugineum] |
| Chr1.2348 | pollen-specific protein-like At4g18596 [Eutrema salsugineum] |
| Chr1.2349 | hypothetical protein AXX17_AT4G21850 [Arabidopsis thaliana] |
| Chr1.2350 | replication protein A 14 kDa subunit B [Eutrema salsugineum] |
| Chr1.2351 | uncharacterized protein LOC18030450 [Eutrema salsugineum] |
| Chr1.2352 | protein CHUP1, chloroplastic [Eutrema salsugineum] |
| Chr1.2359 | uncharacterized protein LOC18030458 [Eutrema salsugineum] |
| Chr1.2360 | PREDICTED: uncharacterized protein At4g18490-like [Camelina sativa] |
| Chr1.2361 | magnesium-chelatase subunit ChlI-1, chloroplastic [Eutrema salsugineum] |
| Chr1.2362 | probable pre-mRNA-splicing factor ATP-dependent RNA helicase DEAH9 [Capsella rubella] |
| Chr1.2363 | probable pre-mRNA-splicing factor ATP-dependent RNA helicase DEAH9 [Eutrema salsugineum] |
| Chr2.3994 | delta(14)-sterol reductase [Eutrema salsugineum] |
| Chr2.3995 | CBS domain-containing protein CBSCBSPB3 [Eutrema salsugineum] |
| Chr2.3996 | peroxiredoxin-2E, chloroplastic [Eutrema salsugineum] |
| Chr2.3998 | zinc finger CCCH domain-containing protein 18 [Eutrema salsugineum] |
| Chr2.3999 | pyruvate kinase 1, cytosolic [Capsella rubella] |
| Chr2.4000 | F-box protein PP2-A15 [Eutrema salsugineum] |
| Chr2.4001 | PREDICTED: probable carbohydrate esterase At4g34215 isoform X1 [Brassica rapa] |
| Chr2.4002 | 60S ribosomal protein L24-2 [Eutrema salsugineum] |
| Chr2.4007 | uncharacterized protein LOC9312253 [Arabidopsis lyrata subsp. lyrata] |
| Chr2.4008 | general transcription and DNA repair factor IIH subunit TFB1-1 [Eutrema salsugineum] |
| Chr2.4009 | beta-galactosidase [Eutrema salsugineum] |
| Chr2.4014 | DEAD-box ATP-dependent RNA helicase 38 [Eutrema salsugineum] |
| Chr2.4015 | PREDICTED: DEAD-box ATP-dependent RNA helicase 38 [Camelina sativa] |
| Chr2.4016 | vacuolar protein-sorting-associated protein 37 homolog 1 [Eutrema salsugineum] |
| Chr2.4017 | carotene epsilon-monooxygenase, chloroplastic [Eutrema salsugineum] |
| Chr2.4023 | transcription factor MYB27 [Eutrema salsugineum] |
| Chr2.4024 | thioredoxin-like 3-3 [Eutrema salsugineum] |
| Chr2.4025 | PREDICTED: cell division control protein 48 homolog D [Camelina sativa] |
| Chr2.4027 | PREDICTED: uncharacterized protein LOC108837042 [Raphanus sativus] |
| Chr2.4028 | PREDICTED: B3 domain-containing protein REM20-like isoform X1 [Raphanus sativus] |
| Chr2.4029 | hypothetical protein EUTSA_v10011019mg [Eutrema salsugineum] |
| Chr2.4030 | PREDICTED: phenylalanine ammonia-lyase 2 [Raphanus sativus] |
| Chr2.4031 | uncharacterized protein LOC18021608 isoform X1 [Eutrema salsugineum] |
| Chr2.4032 | PREDICTED: B3 domain-containing protein REM20-like isoform X2 [Raphanus sativus] |
| Chr2.4033 | uncharacterized protein LOC18021602 [Eutrema salsugineum] |
| Chr2.4034 | nuclear transcription factor Y subunit B-10 [Eutrema salsugineum] |
| Chr2.4057 | PREDICTED: uncharacterized protein LOC108818185 isoform X2 [Raphanus sativus] |
| Chr2.4058 | uncharacterized protein LOC18021478 isoform X1 [Eutrema salsugineum] |
| Chr2.4064 | putative leucine-rich repeat receptor-like serine/threonine-protein kinase At3g53590 isoform X1 [Arabidopsis lyrata subsp. lyrata] |
| Chr2.4065 | PREDICTED: zinc finger protein ZAT11-like [Camelina sativa] |
| Chr2.4416 | PREDICTED: condensin complex subunit 1-like [Brassica oleracea var. oleracea] |
| Chr2.4417 | uncharacterized protein At3g28850 [Eutrema salsugineum] |
| Chr2.4418 | DNA-directed RNA polymerase V subunit 5A [Eutrema salsugineum] |
| Chr2.4419 | hypothetical protein BRARA_I03938 [Brassica rapa] |
| Chr2.4420 | PREDICTED: uncharacterized protein LOC106309629 [Brassica oleracea var. oleracea] |
| Chr2.4421 | mitochondrial fission 1 protein A [Eutrema salsugineum] |
| Chr2.4422 | PREDICTED: origin of replication complex subunit 5 isoform X1 [Camelina sativa] |
| Chr2.4423 | eIF-2-alpha kinase GCN2 isoform X4 [Populus trichocarpa] |
| Chr2.4424 | lysM domain receptor-like kinase 3 [Eutrema salsugineum] |
| Chr2.4425 | regulatory protein NPR6 [Eutrema salsugineum] |
| Chr2.4433 | UDP-N-acetylglucosamine--dolichyl-phosphate N-acetylglucosaminephosphotransferase [Eutrema salsugineum] |
| Chr2.4434 | PREDICTED: glucan endo-1,3-beta-glucosidase, acidic isoform-like [Brassica oleracea var. oleracea] |
| Chr2.4435 | hypothetical protein AALP_AA5G216400 [Arabis alpina] |
| Chr2.4436 | probable glucan endo-1,3-beta-glucosidase BG1 [Eutrema salsugineum] |
| Chr2.4437 | protein FATTY ACID EXPORT 1, chloroplastic-like [Brassica napus] |
| Chr2.4438 | hypothetical protein EUTSA_v10005924mg [Eutrema salsugineum] |
| Chr2.4439 | DNA helicase INO80 [Eutrema salsugineum] |
| Chr2.4440 | DNA helicase INO80 [Eutrema salsugineum] |
| Chr2.4441 | uncharacterized protein LOC18019964 [Eutrema salsugineum] |
| Chr2.4442 | putative calcium-transporting ATPase 11, plasma membrane-type [Eutrema salsugineum] |
| Chr2.4443 | putative calcium-transporting ATPase 11, plasma membrane-type [Eutrema salsugineum] |
| Chr2.4444 | chaperone protein dnaJ 49 [Eutrema salsugineum] |
| Chr2.4448 | agamous-like MADS-box protein AGL18 [Eutrema salsugineum] |
| Chr2.4449 | uncharacterized protein LOC18019956 [Eutrema salsugineum] |
| Chr2.4450 | villin-3 [Eutrema salsugineum] |
| Chr2.4452 | hypothetical protein EUTSA_v10005782mg [Eutrema salsugineum] |
| Chr2.4453 | hypothetical protein AALP_AA5G218600 [Arabis alpina] |
| Chr2.4454 | uncharacterized protein LOC18019872 [Eutrema salsugineum] |
| Chr3.831 | bZIP transcription factor 17 [Eutrema salsugineum] |
| Chr3.832 | PLAC8 family protein [Arabidopsis thaliana] |
| Chr3.833 | exocyst complex component SEC15A [Capsella rubella] |
| Chr3.834 | ubiquitin carboxyl-terminal hydrolase 5 [Arabidopsis lyrata subsp. lyrata] |
| Chr3.848 | PREDICTED: probable RNA-binding protein EIF1AD [Camelina sativa] |
| Chr3.849 | E3 ubiquitin-protein ligase SHPRH [Eutrema salsugineum] |
| Chr3.850 | E3 ubiquitin-protein ligase SHPRH [Eutrema salsugineum] |
| Chr3.851 | hypothetical protein BRARA_D02485 [Brassica rapa] |
| Chr3.852 | rhodanese-like domain-containing protein 7 [Eutrema salsugineum] |
| Chr3.853 | probable WRKY transcription factor 54 [Eutrema salsugineum] |
| Chr3.854 | hypothetical protein EUTSA_v10017926mg, partial [Eutrema salsugineum] |
| Chr3.855 | N-terminal kinase-like protein [Eutrema salsugineum] |
| Chr3.856 | pentatricopeptide repeat-containing protein At2g40720 [Eutrema salsugineum] |
| Chr6.3117 | hypothetical protein BRARA_I02035 [Brassica rapa] |
| Chr6.3118 | F-box protein SKIP28 isoform X1 [Eutrema salsugineum] |
| Chr6.3119 | glucan endo-1,3-beta-glucosidase 3 [Eutrema salsugineum] |
| Chr6.3120 | putative ribosome biogenesis protein slx9-like [Eutrema salsugineum] |
| Chr6.3121 | plant UBX domain-containing protein 2 [Eutrema salsugineum] |
| Chr6.3122 | cysteine-rich repeat secretory protein 12-like [Brassica napus] |
| Chr6.3123 | PREDICTED: nudix hydrolase 17, mitochondrial [Brassica rapa] |
| Chr6.3128 | dolichyl-diphosphooligosaccharide--protein glycosyltransferase subunit 1B [Eutrema salsugineum] |
| Chr6.3129 | cleavage and polyadenylation specificity factor subunit 3-II isoform X1 [Arabidopsis lyrata subsp. lyrata] |
| Chr6.3130 | E3 ubiquitin protein ligase RIE1 [Eutrema salsugineum] |
| Chr6.3131 | pentatricopeptide repeat-containing protein At2g01740 [Eutrema salsugineum] |
| Chr6.3132 | microtubule-associated protein 70-3 [Eutrema salsugineum] |
| Chr6.3133 | PREDICTED: uncharacterized protein LOC106304120 [Brassica oleracea var. oleracea] |
| Chr6.3134 | LOW QUALITY PROTEIN: two-component response regulator ARR14 [Eutrema salsugineum] |
| Chr6.3135 | vacuolar iron transporter 1 [Eutrema salsugineum] |
| Chr6.3136 | PREDICTED: 40S ribosomal protein S19-3 [Raphanus sativus] |
| Chr6.3144 | purple acid phosphatase 8 [Eutrema salsugineum] |
| Chr6.3145 | PREDICTED: type IV inositol polyphosphate 5-phosphatase 9 [Brassica rapa] |
| Chr6.3146 | cyclin-J18 [Eutrema salsugineum] |
| Chr6.3147 | 65-kDa microtubule-associated protein 6 [Brassica napus] |
| Chr6.3148 | uncharacterized protein BNAC09G20880D [Brassica napus] |
| Chr6.3149 | putative C2H2 zinc finger protein [Eutrema halophilum] |
| Chr6.3150 | serine/threonine-protein kinase BRI1-like 2 [Eutrema salsugineum] |
| Chr6.3155 | BnaA02g26140D [Brassica napus] |
| Chr6.3158 | unnamed protein product, partial [Brassica rapa] |
| Chr6.3159 | putative Myb family transcription factor At1g14600 [Eutrema salsugineum] |
| Chr6.3160 | uncharacterized protein At4g15970 [Eutrema salsugineum] |
| Chr6.3162 | protein indeterminate-domain 4, chloroplastic isoform X1 [Eutrema salsugineum] |
| Chr6.3173 | phytosulfokine receptor 1 [Eutrema salsugineum] |
