# Supplementary material for: Genomic analysis of field pennycress (Thlaspi arvense) provides insights into mechanisms of adaptation to high elevation
Source: BMC Biol. 2021 Jul 22;19:143. doi: 10.1186/s12915-021-01079-0 (PMC8296595; doi:10.1186/s12915-021-01079-0)
Supplement: Supplementary file 18 — Additional file 18: Figure S4. Mapping re-sequenced short reads of four populations to the reference genome and the coverage of the flowering related genes (FRI, FT, CONSTANS, GIGANTEA, CDF, SPL, PIF and TSF) region using IGV. [file 12915_2021_1079_MOESM18_ESM.docx]

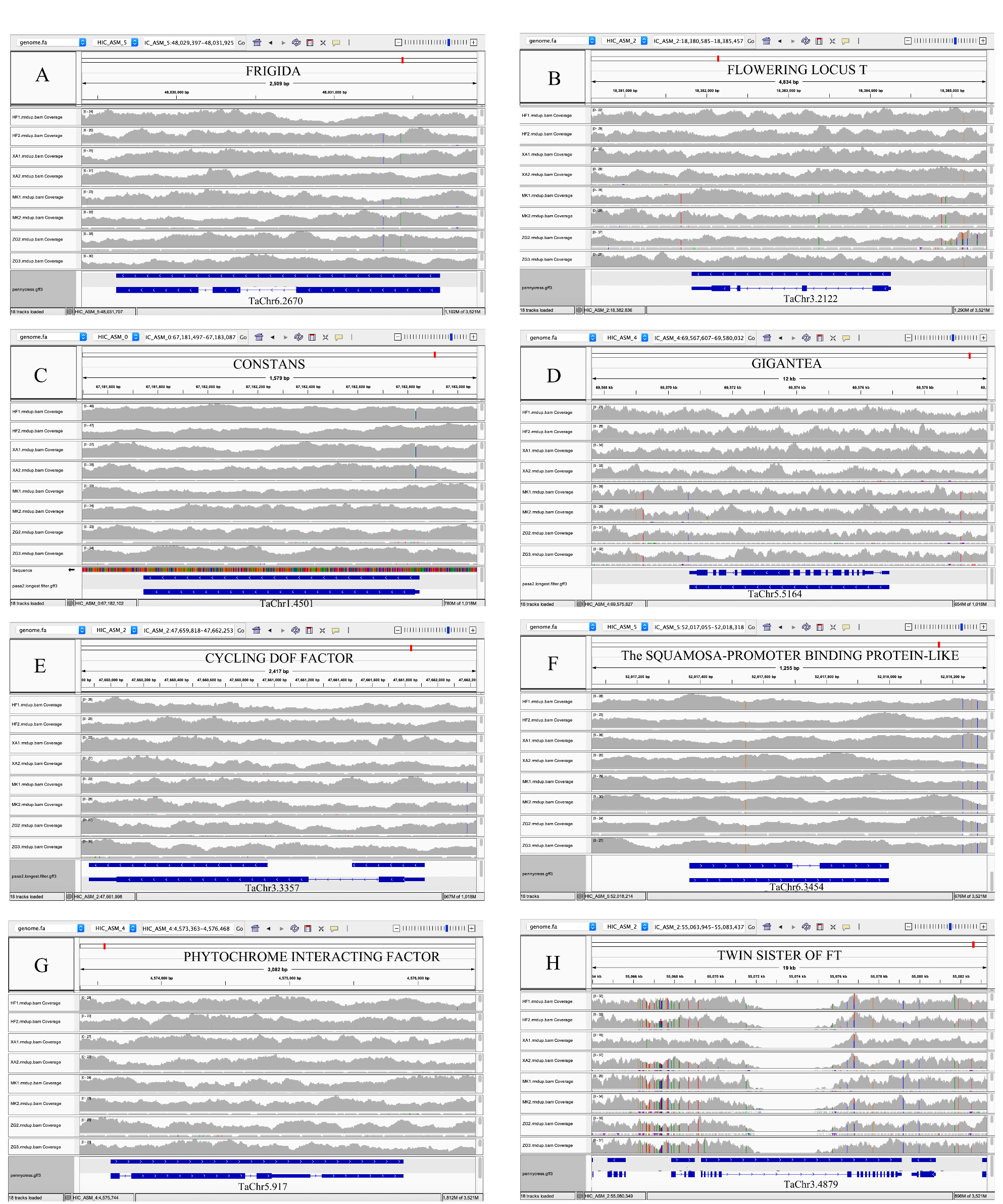


Figure S4. Mapping re-sequenced short reads of four populations to the reference genome and the coverage of the flowering related genes (*FRI*, *FT*, *CONSTANS*, *GIGANTEA*, *CDF*, *SPL*, *PIF* and *TSF*) region using IGV.
