# Supplementary material for: Genomic analysis of field pennycress (Thlaspi arvense) provides insights into mechanisms of adaptation to high elevation
Source: BMC Biol. 2021 Jul 22;19:143. doi: 10.1186/s12915-021-01079-0 (PMC8296595; doi:10.1186/s12915-021-01079-0)
Supplement: Supplementary file 20 — Additional file 20: Figure S6. Mapping re-sequenced short reads of four populations to the reference genome and the coverage of the FLC region using IGV. The red dotted box shows the mutation site (G>C). [file 12915_2021_1079_MOESM20_ESM.docx]

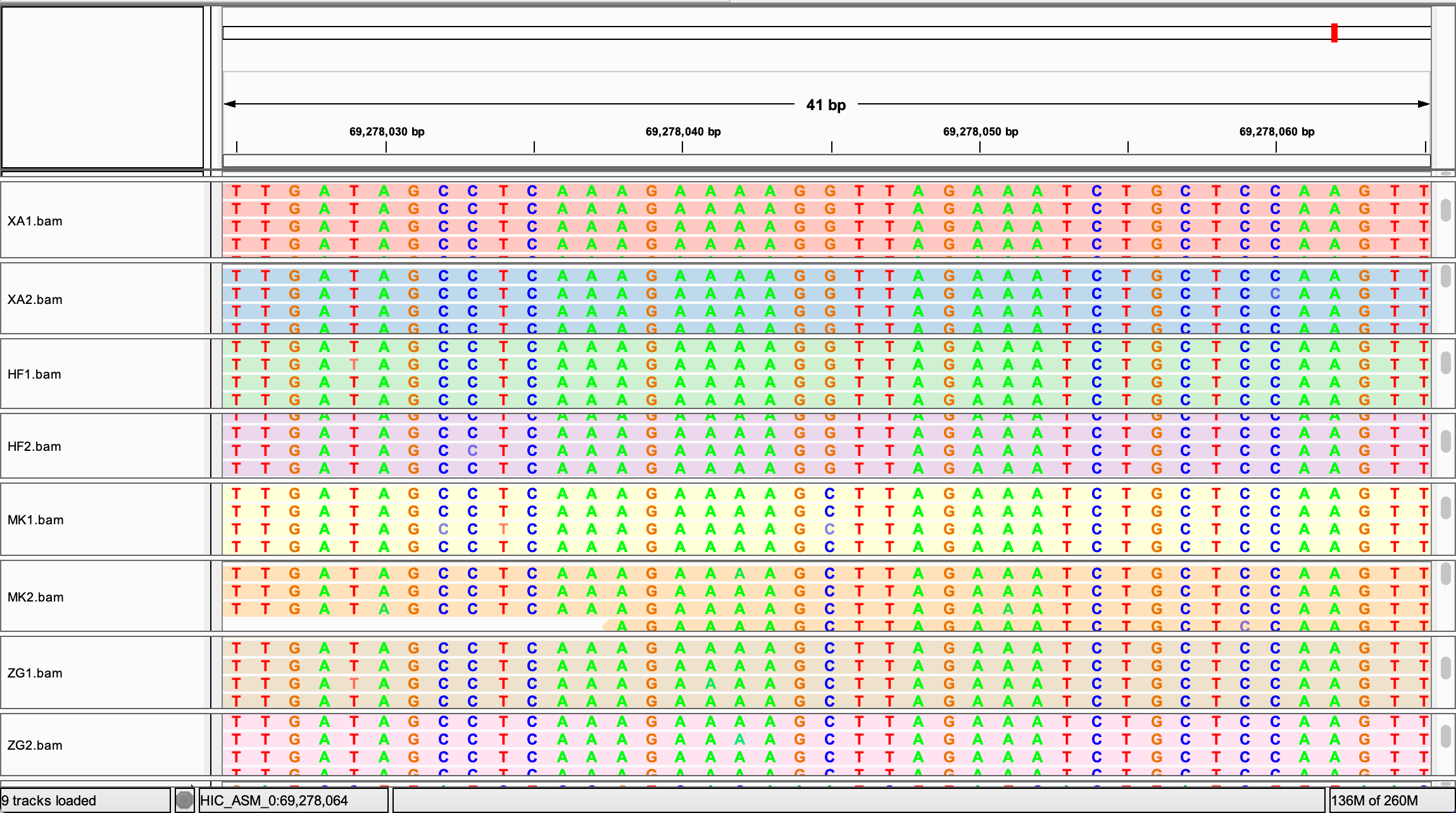


Figure S6. Mapping re-sequenced short reads of four populations to the reference genome and the coverage of the *FLC* region using IGV. The red dotted box shows the mutation site (G>C).
